# Neandertals on the beach: Use of marine resources at Grotta dei Moscerini (Latium, Italy)

**DOI:** 10.1371/journal.pone.0226690

**Published:** 2020-01-15

**Authors:** Paola Villa, Sylvain Soriano, Luca Pollarolo, Carlo Smriglio, Mario Gaeta, Massimo D’Orazio, Jacopo Conforti, Carlo Tozzi

**Affiliations:** 1 Museum of Natural History, University of Colorado, Boulder, Colorado, United States of America; 2 Istituto Italiano di Paleontologia Umana, Rome, Italy; 3 School of Geography, Archaeology and Environmental Studies, University of the Witwatersrand, Johannesburg, South Africa; 4 ArScAn, AnTET, CNRS, Maison de l’Archéologie et de l’Ethnologie, Université Paris Nanterre, France; 5 Laboratoire Archéologie et Peuplement de l’Afrique, University of Geneva, Genève, Switzerland; 6 Dipartimento di Scienze, Università Roma, Roma, Italy; 7 Dipartimento di Scienze della Terra, Università di Roma La Sapienza, Rome, Italy; 8 Dipartimento di Scienze della Terra, Università di Pisa, Pisa, Italy; 9 Dipartimento Civiltá e Forme del Sapere, Università di Pisa, Pisa, Italy; Max Planck Institute for the Science of Human History, GERMANY

## Abstract

Excavated in 1949, Grotta dei Moscerini, dated MIS 5 to early MIS 4, is one of two Italian Neandertal sites with a large assemblage of retouched shells (n = 171) from 21 layers. The other occurrence is from the broadly contemporaneous layer L of Grotta del Cavallo in southern Italy (n = 126). Eight other Mousterian sites in Italy and one in Greece also have shell tools but in a very small number. The shell tools are made on valves of the smooth clam *Callista chione*. The general idea that the valves of *Callista chione* were collected by Neandertals on the beach after the death of the mollusk is incomplete. At Moscerini 23.9% of the specimens were gathered directly from the sea floor as live animals by skin diving Neandertals. Archaeological data from sites in Italy, France and Spain confirm that shell fishing and fresh water fishing was a common activity of Neandertals, as indicated by anatomical studies recently published by E. Trinkaus. Lithic analysis provides data to show the relation between stone tools and shell tools. Several layers contain pumices derived from volcanic eruptions in the Ischia Island or the Campi Flegrei (prior to the Campanian Ignimbrite mega-eruption). Their rounded edges indicate that they were transported by sea currents to the beach at the base of the Moscerini sequence. Their presence in the occupation layers above the beach is discussed. The most plausible hypothesis is that they were collected by Neandertals. Incontrovertible evidence that Neandertals collected pumices is provided by a cave in Liguria. Use of pumices as abraders is well documented in the Upper Paleolithic. We prove that the exploitation of submerged aquatic resources and the collection of pumices common in the Upper Paleolithic were part of Neandertal behavior well before the arrival of modern humans in Western Europe.

## Introduction

**Fig 1:**
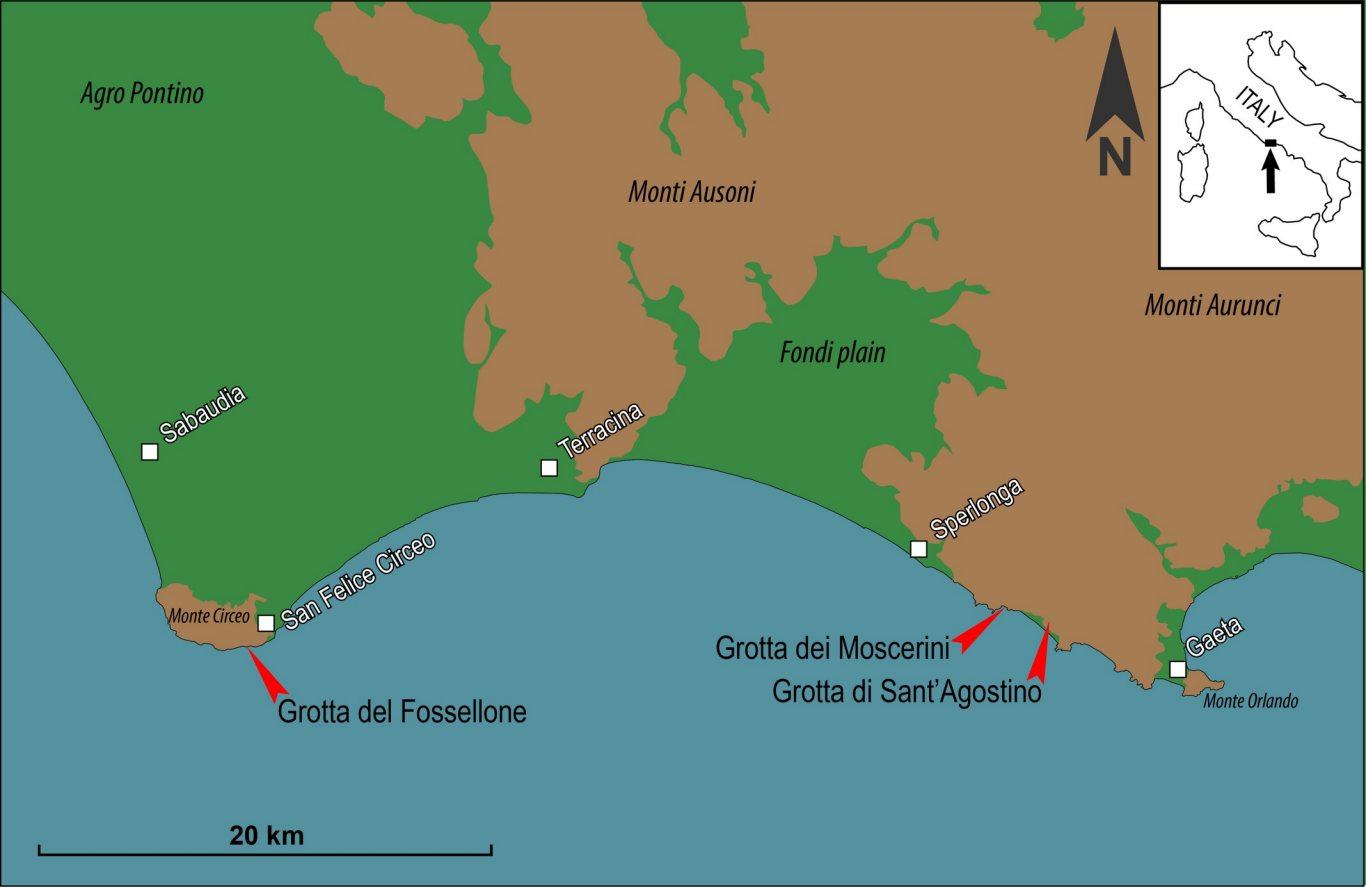
Map showing the location of Grotta dei Moscerini (41°14’21.01” N; 13°28’42.16” E), Grotta del Fossellone (41°13’26.29” N; 13°04’50.53” E) and Grotta di Sant’Agostino (41°14’01” N; 13°30’13.08” E).

Moscerini is one of the largest coastal caves in the Latium, between Sperlonga and Gaeta and about 5 km from Grotta Sant’Agostino [1]. The site is located at the base of a limestone cliff on the south-eastern flank of a small coastal promontory (Fig 1).

It was discovered during a 1936–38 survey of coastal caves from Rome to Gaeta by A.C. Blanc, A. Segre and co-workers of the Italian Institute of Human Paleontology. It was excavated in August 1949 under the supervision of A.C. Blanc [2]. A 2 m. by 1.5 m. trench was established on the deposits behind the dripline of the cavity. Since the main excavated area is small (only 3 sq m. representing 5% of the total preserved site area of 56 sq m.) the assemblage sizes for individual layers are also small.

The cave is close to the modern shoreline and the base of the stratigraphic section is about 3 m asl [2: fig 3.14]. The almost 9 m thick sequence was drawn and described by A. Segre in 1949 and divided into geological layers numbered (top to bottom) from 1 to 44. The archaeological materials were recovered and labelled according to these layers. According to photos taken during the excavation [2: fig 3.19 D] dry sieving with a relatively small (5 or 10 mm) mesh was used suggesting that there are minor recovery biases.

**Fig 2:**
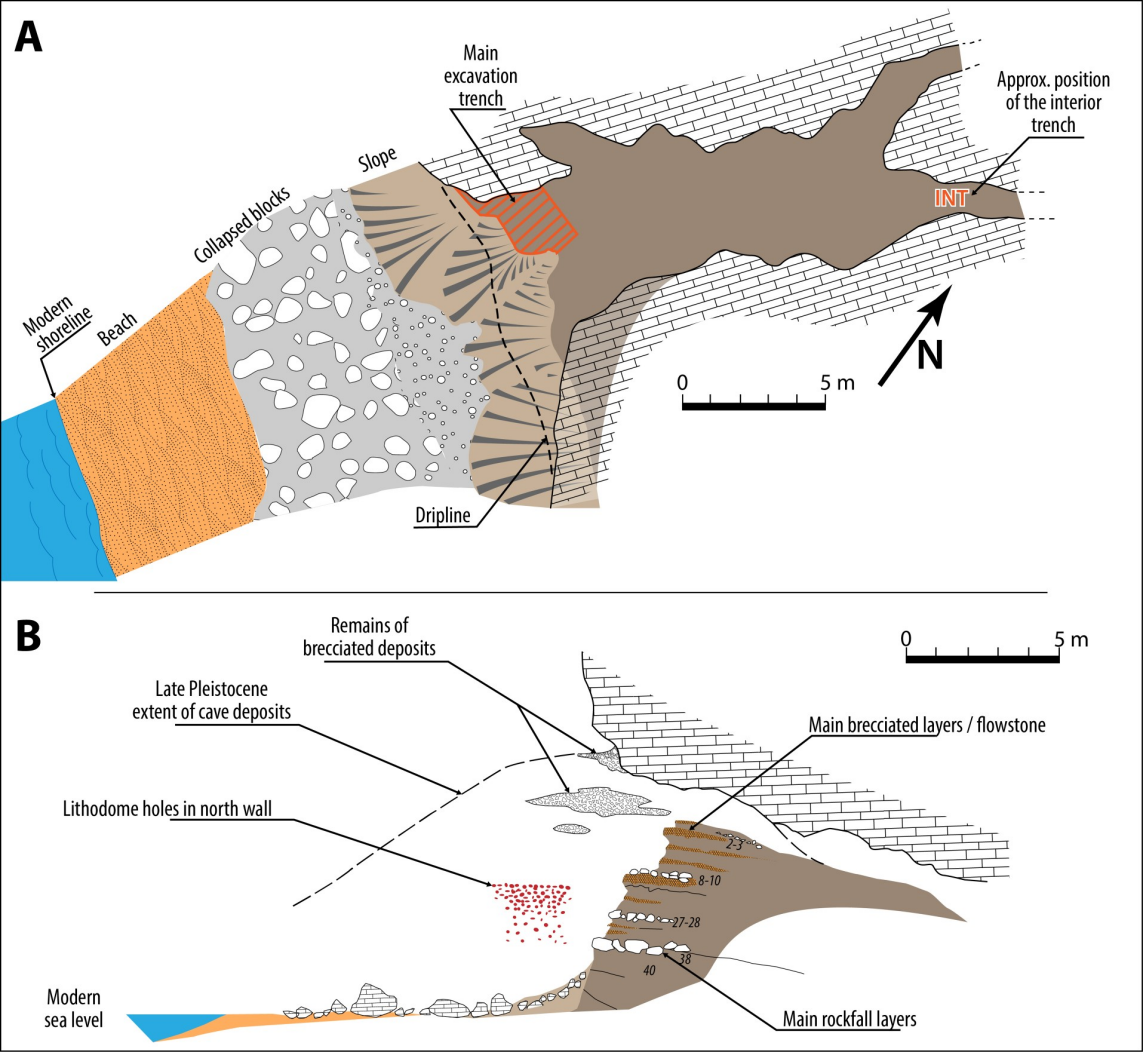
(A) Plan view of Grotta dei Moscerini. (B) Cross-sectional view of the cave with estimated extent of cultural deposits prior to the Holocene sea rise. The numbers correspond to layers in Fig 3. Drawing by Sylvain Soriano based on field drawings by the late Aldo Segre.

A test pit was opened inside the cave approximately 15 meters away from the modern entrance (Fig 2A). It was excavated by arbitrary levels (labelled Interno 1 to Interno 4, from the top to the base). It is impossible to correlate the main section and the internal trench but according to [3] it is likely that these levels correspond with the latest layers from the main section. If we take into account the gentle dipping of layers toward the interior of the cave, the layers from the internal test pit might be even (partly) younger.

The site is no longer accessible because it was buried in early 1970s under rocks and boulders blasted from the side of the hill during construction of the coastal highway (Via Flacca). Thus none of the scholars who first studied fauna or industries [2–4] were able to enter the site.

The top of the sequence is sealed by a 25 cm thick stalagmite horizontal layer (layer 2). The lowermost layer (layer 44) is a sandy deposit rich in marine molluscs. *Strombus bubonius* was not seen by [2] but was recorded in Segre’s field notes. Its lowest level also had many yellow and light grey pumices and some unretouched pebbles of 4–5 cm in diameter. It is interpreted as a sandy beach deposit and considered to date from the Last Interglacial. Layer 42 is described by Segre as a volcanic tuff. Layer 41 is the first archaeological layer with a stalagmitic lens at the base and few stone artifacts (N = 7).

Many layers are sandy with small angular clasts; two layers (41–40) consist of sandy-clay fill with rare pebbles (4 and 8 cm in diameter respectively). Four episodes of rock fall are documented in layer 3, layers 8–9, layers 27–28 and layer 38. Excavation photos in Stiner [2: 3.14; 3.19 A] show many fissures in the cave vault, probable cause of the rock falls present in the deposits. A wave-cut notch and several rows of lithodomes i.e. holes formed and inhabited by bivalves that live mainly in the area battered by waves (*Lithophaga lithophaga*, Linnaeus 179*8*) present on the north wall (Fig 2B) at approximately the level of layers 8–20 [2: p. 47] and are thought to mark the maximum level of the sea corresponding to MIS 5.5. At that time the cave was probably free of continental deposits, totally washed out by the high sea level. Lithodome scars were also noted by Segre on the large limestone blocks of layer 38, most likely fallen from the cave wall.

**Fig 3:**
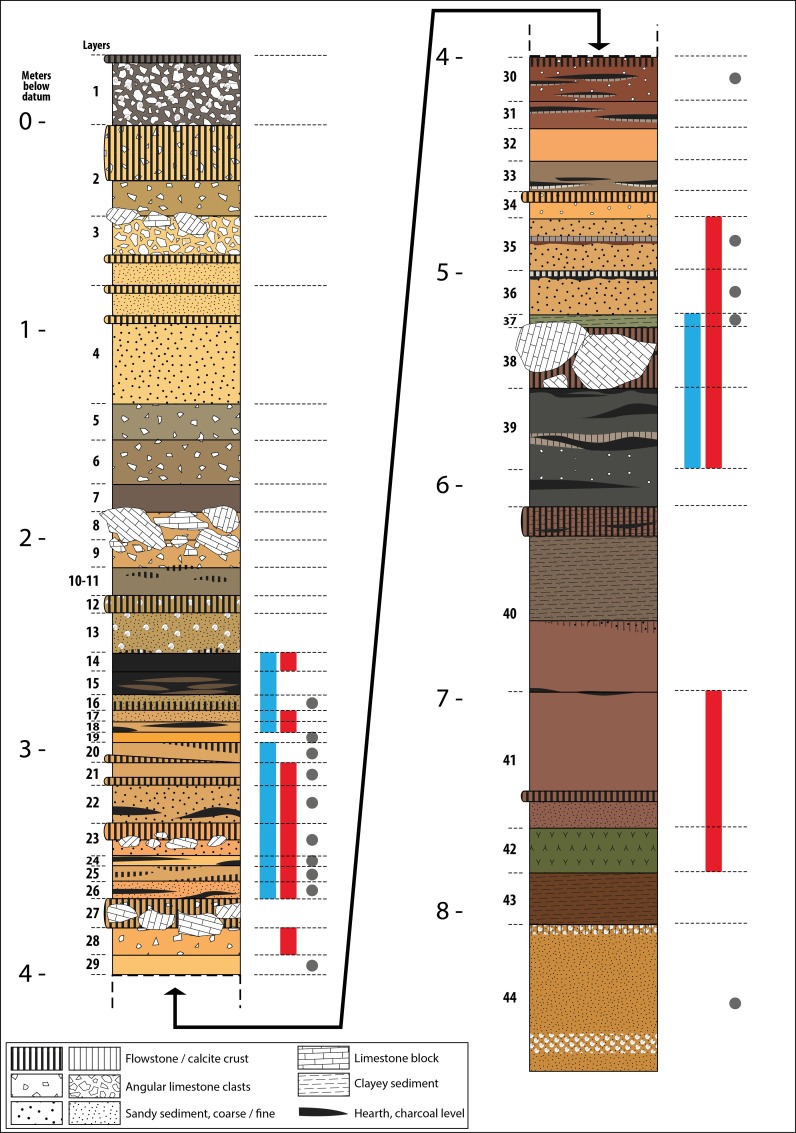
Stratigraphic section of the main excavation area at Grotta dei Moscerini. Numbers to the left are meters below datum, numbers in smaller font are layers. Red shading indicates all layers with the retouched shells described in the present paper. Blue shading corresponds to layers of which the lithic industry was analyzed by us. The grey dots mark layers that contained one or more pumices. Based on Segre’s observations, layer 16, layers 35–36 and especially layer 44 contained pumices but we did not find these pumices in the material stored at Anagni. Drawing by Sylvain Soriano based on field drawings and information provided by Aldo Segre, courtesy of Aldo Segre.

Artifacts are present throughout the sequence from layer 41 up to layer 14 but they become rare upward to layer 1. Evidence of fire (charcoal and ashes, sometimes burnt bones) occur in almost all layers (layers 11, 14–16, 18–19, 22–26, 29–33, 35, 36, 38–41). Yellow and light grey pumices occur in several levels (grey dots in Fig 3).

### Dating

An ESR chronology from Moscerini was published in 1991 by [5] together with amino-acid chronology (AAR) measurements on molluscs. As the site was no longer accessible for additional sampling, small remains of sediments adhering to some sampled teeth provided U, Th and K content for the estimation of dose rate. Thus error ranges are not listed. Red deer teeth and one hippopotamus tooth from layers 39, 38, 35, 33, 26 and 25 were analyzed. Except for those from layers 35 and 25, two sub-samples were measured for each tooth. In the absence of *in situ* measurement of external dose rate, these ESR dates must be considered cautiously and cannot support any detailed interpretation of the chronology of the archaeological sequence. The lower part of the sequence might have accumulated during MIS 5, in agreement with [5] correlation of the basal sandy beach deposits (layer 44) with the Last Interglacial supported by AAR (amino acid geochronology). The upper part might date to the end of MIS 5 or beginning of MIS 4. Table 1 shows the dates obtained using the Linear Uptake (LU) results since the Early Uptake model yields what may be considered minimum ages [2].

**Table 1:** Grotta dei Moscerini, ESR dates for tooth enamel (after Schwarcz et al.1990-1991 and Stiner 1994).

Layers	Material	Date (ka)	Average of two samples
25	red deer tooth	79	
26	red deer tooth	67	74 ± 7
26	red deer tooth	81	
33	red deer tooth	89	106 ±17
33	red deer tooth	123	
35	red deer tooth	66	
38	red deer tooth	96	101 ± 5
38	red deer tooth	106	
39	red deer tooth	96	96 ± 1
39	Hippopotamus tooth	97	

For a detailed reconstruction of southern Latium sea levels during the Last Interglacial see [6].

## Materials and methods

### Repositories and sampling

#### Lithics

The bulk of the lithic assemblage from Grotta dei Moscerini (N = 1139, excluding artifacts without stratigraphic provenience, labelled *Ripulitura scavo*, *Ripulitura esterna* or *Esterno*) is housed at the Italian Institute of Human Paleontology in the town of Anagni (Latium) whereas a small sample (N = 85) is stored and partly exhibited in the Pigorini National Museum of Prehistory and Ethnography in Rome. Of the 85 artifacts at the Pigorini Museum only 35 could be fully analysed; the rest of the pieces were in showcases, hence not available for detailed analysis. All modified shells were studied whereas we selected only the lithic artifacts coming from layers containing modified shells (Fig 3). Layers 15, 16 and 20 where retouched shells are missing were added for stratigraphic continuity of data. With the exception of Interno 1, the highest and youngest level in the interior test pit, lithics from the lower levels of the test pit were not studied due to lack of correlation with the main excavation area.

Differently from our previous studies of a Middle Paleolithic (MP thereafter) industry from Latium [7], the sorting threshold was lowered to 10 mm instead of 15 mm to enlarge our sample. Flake fragments without platform, flakes <1 cm and chunks were excluded from analysis. All cores, core fragments, tools, tool fragments were selected regardless of size.

#### Shell tools

Retouched *Callista chione* are found in many levels of Moscerini, from 42 to 14, for a total of 171 specimens. This large assemblage, including unretouched fragments, is housed in the Italian Institute of Human Paleontology in the town of Anagni, with 17 retouched specimens kept in the Pigorini Museum of Prehistory and Ethnography in Rome.

We examined all shells of *Callista* and extracted the retouched pieces, adding several specimens to the bag containing retouched pieces selected by previous workers. All the unretouched pieces in the archaeological layers, that is excluding layer 44 and 43 which are the beach levels at the base of the sequence, are heavily fragmented. We did not count the unretouched pieces due to the difficulty of distinguishing *Callista* from *Glycymeris* on fragments without the hinge, as noted by Stiner who provides a total of 1230 unretouched and retouched *Callista* and *Glycymeris* fragments summed together [2: table 6.12]. However using the ratio of total hinge specimens of *Callista* (n = 182) versus Glycymeris (n = 85) provided by Stiner [2: table 6.13] we can say that about 68% of the 1230, i.e. about 836 specimens are *Callista* fragments. This is a plausible estimate of the total retouched and unretouched *Callista chione* specimens in the archaeological layers.

#### Problems of species identification

**Fig 4:**
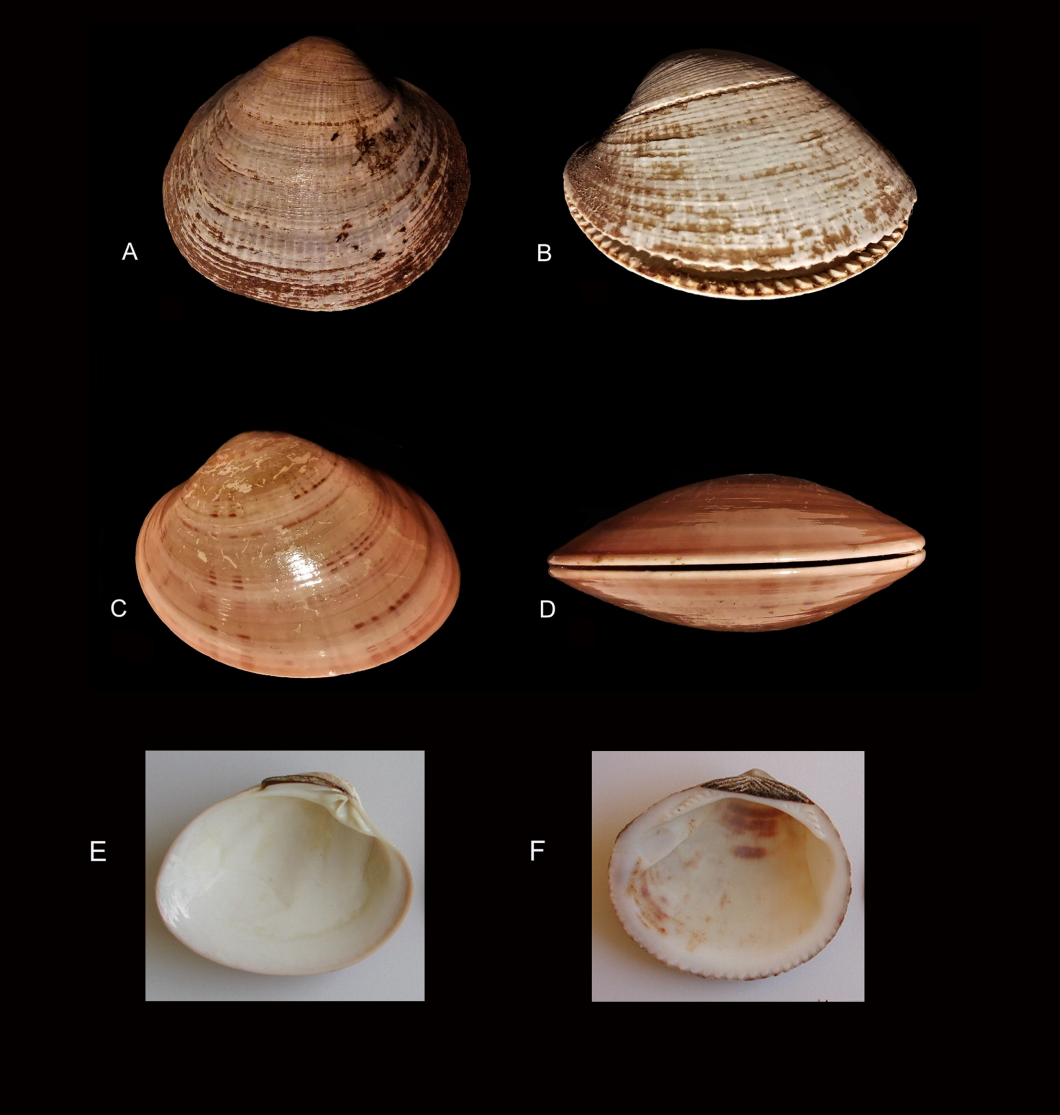
(A-B) Glycymeris; (C-D) Callista chione; (E) internal face of Callista chione; (F) internal face of Glycymeris. A-D photos by Carlo Smriglio. E-F photos courtesy of Barbara Wilkens (permission in S4 File).

Two features help in distinguishing the two species: 1. the distal edge (fringe) of *Callista chione* is smooth but it is indented in *Glycymeris* (Fig 4B and 4D); 2. the different morphology of the hinge (Fig 4E and 4F). Fragments that lack these portions of the shell are difficult to identify. The external surface of valves in *Glycymeris* is marked by radial lines from the umbo to the distal edge; in *Callista* the radial lines are so thin that the surface appears smooth and shining. However on beached specimens or specimens that have been altered (encrusted, abraded) in the cave these differences are no longer easy to see. Thus the species identification on shell fragments that lack the hinge or an intact distal edge is uncertain and may require microscopic analysis. All retouched shells were analyzed under a microscope by Carlo Smriglio and their identification as *Callista* is sound.

#### Pumices

Layers 16 to 37 and layers Interno 1 to 4 yielded 49 pumices or pumice fragments. They are kept in the storage area of the Italian Institute of Human Paleontology in the town of Anagni (Latium). Level provenience was written on paper labels; we added an inventory number and bagged each in individual plastic Minigrips.

### Permits

Permits to study and take photos of the materials were obtained from the President of the Italian Institute of Human Paleontology (Fabio Parenti and more recently Stefano Grimaldi) and from the Soprintendenza of the Pigorini Museum (Prot. N. 2408, 25.02.03/15 and MBAC-S-MNPE Pigorini Cl. 23.03.02/3).

### Methods

The study of the lithic and shell assemblages is based on quantitative and qualitative analyses, including metrical, taphonomic, technological and typological attributes, as described in previous publications [7: S5 File]. We coded all specimens in Excel files. Photographs were taken with a Nikon D 800. The description of the lithic industry (text, tables and figures) is provided in the S2 File.

**Fig 5:**
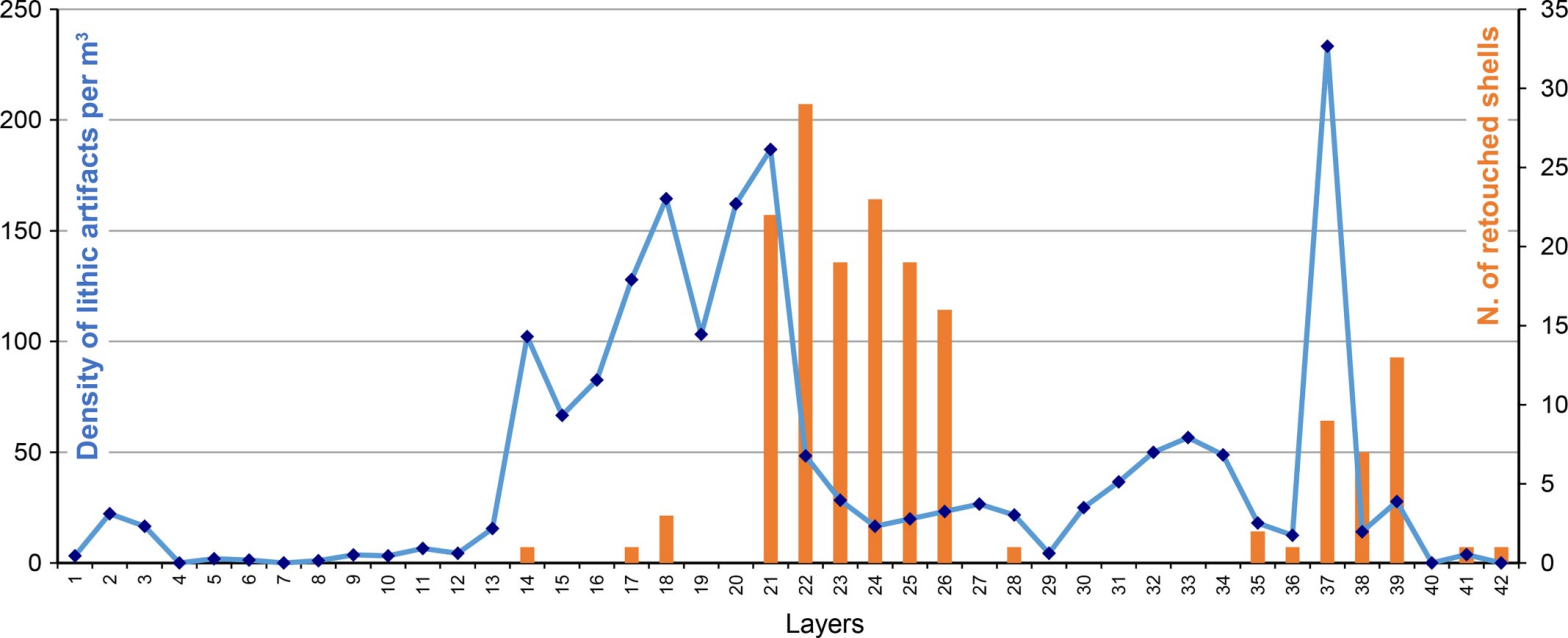
**Variations of density of lithic artifacts per layer (in blue) at Moscerini cave in the main excavation compared to the number of retouched shells per layer (in orange).** Lithic pieces with broad provenience (layers 1–5, N = 4; layers 12–17, N = 36) where not included here. The internal test pit (layers Int 1. to Int. 4) is excluded from the analysis since the size of its excavated surface is unknown.

The low density of lithic finds in almost all layers forced us to group layers into three sets for the study, as done by Kuhn [3]. Layer INT 1 was included because it is the youngest layer of the series [3: p. 61]. Criteria for grouping layers are exposed in the S2 File p.3, and S2 File, Fig 5A. Tables detailing the technological composition of the assemblage for each layer are also in S2 File (Table 2A and 2B and Table 3A and 3B). Technological differences (frequencies of cores and flake types) between assemblages from each later are minimal and no significant changes can be seen on these grounds. Thus we choose to indicate the general assemblage composition in Table 2, and to seek the technological and techno-economical differences through the use of significant ratios, as in S2 File, Fig 5B–5H. In the main text lithic analysis is used primarily to explore the significance of recycling, reworking and use of patinated blanks in relation to shell tools (see”Relationship of stone and shell tools”).

**Table 2:** Grotta dei Moscerini. Lithic assemblage composition (Layers 39–37, 26–20, 18–14 and INT1).

	Unretouched		Retouched		Total	
Categories	N	%	N	%	N	%
Flakes and flake fragments	220	54.6	183	45.4	403	100
Cores and core fragments	35	55.6	28	44.4	63	100
Chunks	n/an/an-		7		7	
Pebble or pebble frag.	2		18		20	
With double patina	1		26		27	
Undetermined blank	n/a		13		13	
Total	258		275		533	
Hammerstone, retoucher on limestone pebble	2					

n/a = not available. Detailed technological composition by layer can be found in S2 File Tables 2A, 2B, 3A, 3B and 5.

**Table 3:** Grotta dei Moscerini, *Callista chione* assemblage.

Levels	Undetermined	Utilized?	Retouched
14			1
17			1
18			3
21			22
22	2	1	29
23			19
24			23
25			19
26	4		16
28			1
35			2
36			1
37			9
38			7
39			13
41			1
42			1
25?			1
INT			1
INT 3			1
**Total**	**6**	**1**	**171**

The petrological analysis of the Moscerini pumices providing information on their texture and glass composition are discussed in the section “Origin of the Moscerini cave pumices”. Their mineralogical composition indicates a provenience from the Campanian volcanoes. Analytical procedures are described in the S1 File. Similar methods were used for the major and trace-element composition analysis of a pumice from a Middle Paleolithic cave in northern Italy (Grotta di Santa Lucia), see section “Analysis of the Santa Lucia pumice” and specific details of techniques in the S1 File.

## Results

### Lithic analysis

The composition of our sample is indicated in Table 2.

### Raw material

#### Procurement

Raw materials are more diversified at Moscerini than in other lithic industries from Latium we have previously studied [7–9]. As in most if not all MP sites from the Latium [3] the main local raw material is small flint pebbles that have been almost exclusively collected in secondary position in beach deposits or fluvial stream or terraces (Figs 1: 3–4, 6–8 in S2 File). The higher roundness of the flint pebbles at Moscerini than at Sedia del Diavolo/Monte delle Gioie [8] points to an origin in beach deposits (active or fossil) rather than fluvial deposits or riverbed [10]. In agreement with such a provenience, 89.0% of cortical surfaces on local flint are rolled or abraded (Table 1 in S2 File). Chert, (silicified) limestone and quartzite are much less frequent but they were collected on the same type of outcrop. A non-local origin is hypothesized for at least three types of flint: a grey-blue chalcedony-like flint (Fig 1: 1 in S2 File), a light brown to dark brown semi-translucent flint (Fig 1: 2, 5 in S2 File) and a grey to black, translucent or semi-translucent flint. Sources of these types of flint are still unknown. Within these non-local raw materials, pieces with abraded or rolled cortex are rare (3.2%) so most of the procurement was made on primary or sub-primary outcrops. This is a major difference with local flint from beach deposits. For a detailed discussion on non-local raw material see S2 File, pp. 3–4.

#### Double patina

Raw material procurement includes pieces with double patina. They are patinated or altered blanks with unpatinated retouch (N = 24) or debitage from patinated/altered cores (N = 3) (Fig 2: D and Fig 3: D in S2 File). Both local (N = 24) and non-local flint (N = 3) were collected in this condition. Collection and use of older, patinated blanks is now commonly described in MP industries [11–16]. The use of blanks with double patina is not a marginal phenomenon at Moscerini as they represent 6.5% of the retouched tools although a higher frequency was observed in the industry from Fossellone layer 23 alpha [15.5% of retouched tools on blanks with double patina) [9].

#### Retouch, reduction and recycling

Frequency of retouched pieces at Moscerini in our sample is the highest we have observed in our previous studies of the MP from the Latium (Table 4 in S2 File). Cores were as frequently retouched as flakes (44.4% of cores *vs*. 45.4% of flakes) suggesting that the selection of blanks to be retouched was not especially determined by the technological status of the blank. This can explain why flint pebbles, fragments of pebbles or chunks have been sometimes directly retouched or shaped bifacially to obtain scrapers (9.1% of retouched tools) (Figs 2C, G; 3I; 4A, in S2 File). Due to reduction of retouched tools, the identification of the blank from 13 retouched tools was impossible and 21 flakes were so much retouched that the type of flake was unidentifiable. Reduction of retouched tools is sometimes evidenced by discrepancies between the final size of the tool and remaining flaking features of the blank itself. In two cases (Figs 2F and 4M in S2 File) we have noticed a very large diameter of the impact point on the platform (~7mm) whereas these retouched tools are less than 20 mm wide. As Hertzian cone fracture developed at the margin of the contact point of the hard hammer [17–18] the diameter of the impact point reflects the size of the hammer. Thus we can conclude that the size of these two blanks before retouch was at least three to four times larger.

The frequency of retouched tools on cores, pebbles or chunks (19.3% of retouched tools; Table 5 in S2 File) is high. It is the same in the Early MP industry from level *d* at Torre in Pietra (19.8%) [7] but it is less frequent at Sant’Agostino layer A1 (9.7%) and at Fossellone layer 23 alpha (3.2%). Like the use of patinated blanks, recycling is recorded all along the stratigraphic sequence [for recycling at Grotta del Cavallo see 15]. Kombewa flakes (flakes from flaked-flakes on ventral surface; 5.2% of flakes, retouched or not) are more frequent than in other MP of the area we studied (Fossellone layer 23 alpha: 2.3%; Sant’Agostino layer A1: 0.9%; Torre in Pietra level d: 1.4%). Recycling includes also the production of flakes on ventral face of retouched tools (Fig 4: C in S2 File) or on dorsal face of flakes or retouched flakes (Nahr-Ibrahim flaking) (Fig 4: G in S2 File) [19–20]. This type of recycling represents 3.0% of flakes, retouched or not, but exotic flint was three times more frequently recycled this way than local flint. The sieving mesh used in the excavation was too large to collect all the very small flakes from tool making but some large ones (N = 20) have been identified (Fig 4: E in S2 File).

### Relationship of stone and shell tools

Fig 5 shows that shell tools are unevenly distributed throughout the stratigraphic sequence at Moscerini so one can wonder if their manufacture and use can be a response to changes in lithic techno-economy, including raw material procurement. Does the use of shell tools correspond to a decrease in stone tools production and/or increase in the intensity of stone tool use (increase of retouched tools, recycling)?

The density of lithic artifacts per cubic meter (n/m^3^) that can be used as a proxy of the intensity of site occupation varies greatly throughout the stratigraphy (Fig 5; Table 7 in S2 File). In most of the layers density is low or very low, especially in the top of the sequence, from layer 13 upward. In more than half of the layers, the density is equal or lower to 20 pieces per m^3^. Compared to other European MP cave sites such as Geissenklösterle (16 to 109 lithic artifacts/m^3^ in MP layers), Hohle Fels (33 to 703 artifacts/m^3^) [21] or Orgnac 3 (48 to 1974 lithic artifacts/m^3^) [22], densities at Moscerini are low, except in layers 18, 20, 21 and 37, suggesting that occupations of the cave were very discontinuous and/or low intensity. For sure only a small area of the cave has been excavated and the frontal part of the deposits had been eroded by the rising of the Holocene sea (Fig 3B) [2 p. 46]. Even so the amount of fallen blocks is not enough to suggest that the greater part of the deposits had been removed. The cubic density of the remains is very low for a cave and it does suggest discontinuous and low intensity occupations.

The density of lithics varies throughout the stratigraphy but it determines successive phases with unequal occupation intensity of the cave, the highest being from layers 21 to 14. Due to the small sample of stone tools from each layer we merged adjacent levels in four phases (Fig 5 in S2 File) to emphasize changes throughout the sequence. Phasing was done with respect to variations in the amount of lithic and shell tools in layers.

Shell tools occur mainly in layers where the density of lithic remains is low; this is generally interpreted to mean that the frequency of shell tools is a response to raw material shortage. According to [23–25] shell tools were a response to poor availability of lithic raw material. It is in layers 26 to 22 that there is the highest frequency of shell tools and stone tools are less abundant. In this phase II, stone tools are very retouched (Fig 5: C in S2 File) and there is low evidence of tool reworking or recycling (Fig 5: G in S2 File) a fact also normally interpreted as due to a shortage of raw material and opportunistic reuse of older stone tools. But local flint from beach deposits is present indicating that the location of the outcrops was known but they were not intensively exploited. The highest frequency of double patina and the highest frequency of non-local flint, suggests that Neandertals of phase 2 arrived with a set of tools from afar and that at Moscerini they collected lithic resources in a very opportunistic way and at very close range. They may have moved to Moscerini cave for specialized tasks linked to coastal resources. In the Upper Paleolithic shell fishing was done most often in winter [26]. It seems that limited activities and ephemeral seasonal occupations are the reasons for the abundance of shell tools. Simply put, the cave has a long stratigraphic sequence but the human occupations were of short duration.

## The shell tool assemblage

Shell tools are made exclusively on *Callista chione* (Linnaeus 1758) a smooth clam of the Veneridae family, which lives on the coasts of the whole Mediterranean Sea and on the coasts of the eastern Atlantic from the British Isles to Morocco [24, 27]. The marine mollusk is edible and is now commercially exploited in Portugal, Spain, France, Italy and Greece [28]. It inhabits soft sandy sea floors in coastal waters ranging from -1 to -180 m, it can reach up to 10 cm in length and it burrows close to the sediment-water interface. The shell syphons reach up to the surface of the sediment so the animal is able to feed, excrete and reproduce. Thus the shell presence under the sand can be perceived.

Implements made on *Callista chione* are known from 11 Mousterian sites of which 10 are in Italy and 1 in southern Greece in Kalamakia Cave [23–24]; see below the section « Shell tools in time and space »). However most sites seem to have very small numbers of retouched *Callista chione* and the implements have not been reported in detail. Until now the best known was Grotta del Cavallo with 126 retouched shells from layer L, analyzed in detail by [24–25].

The Mousterian stratigraphic sequence of Grotta del Cavallo, approximately 4 m thick, dates between MIS 5.5 (129 to 116 ka) [29] at the base (layer N, a Tyrrhenian beach gravel) and 45.5 ± 1.0 ka at the top (layer Fa, a volcanic ash layer; [30]. Layer G in the middle of the sequence is another ash layer dated to 108.7 ± 0.9 ka [30]. Thus layer L below G must date between MIS5.5 and MIS 5.2, and is broadly contemporaneous to the early part of the Moscerini sequence.

Retouched *Callista chione* are found in many levels of Moscerini, from 42 to 14, for a total of 171 specimens (Table 3). Most retouched specimens (n = 128/171) come from levels 21 to 26 representing a deposit of about 1 m thick, rich in charcoal and stalagmite lenses, based on observation by Segre 1949 reported in [2].

*Callista chione* is edible but at Moscerini it seems to have been used mostly to make scrapers. Other kinds of mollusks living in shallow waters (*Cardium*, *Glycimeris*) occur at Moscerini in small quantities but the most common are the Mediterranean mussels (*Mytilus galloprovincialis*) which occur in colonies under water (maximum depth -4-5 m) but adhering to rocks and therefore easier to collect in large quantities (NISP = 1537) [2]. None of these shells were retouched or modified in any way; they were most probably collected for food only [2].

**Fig 6:**
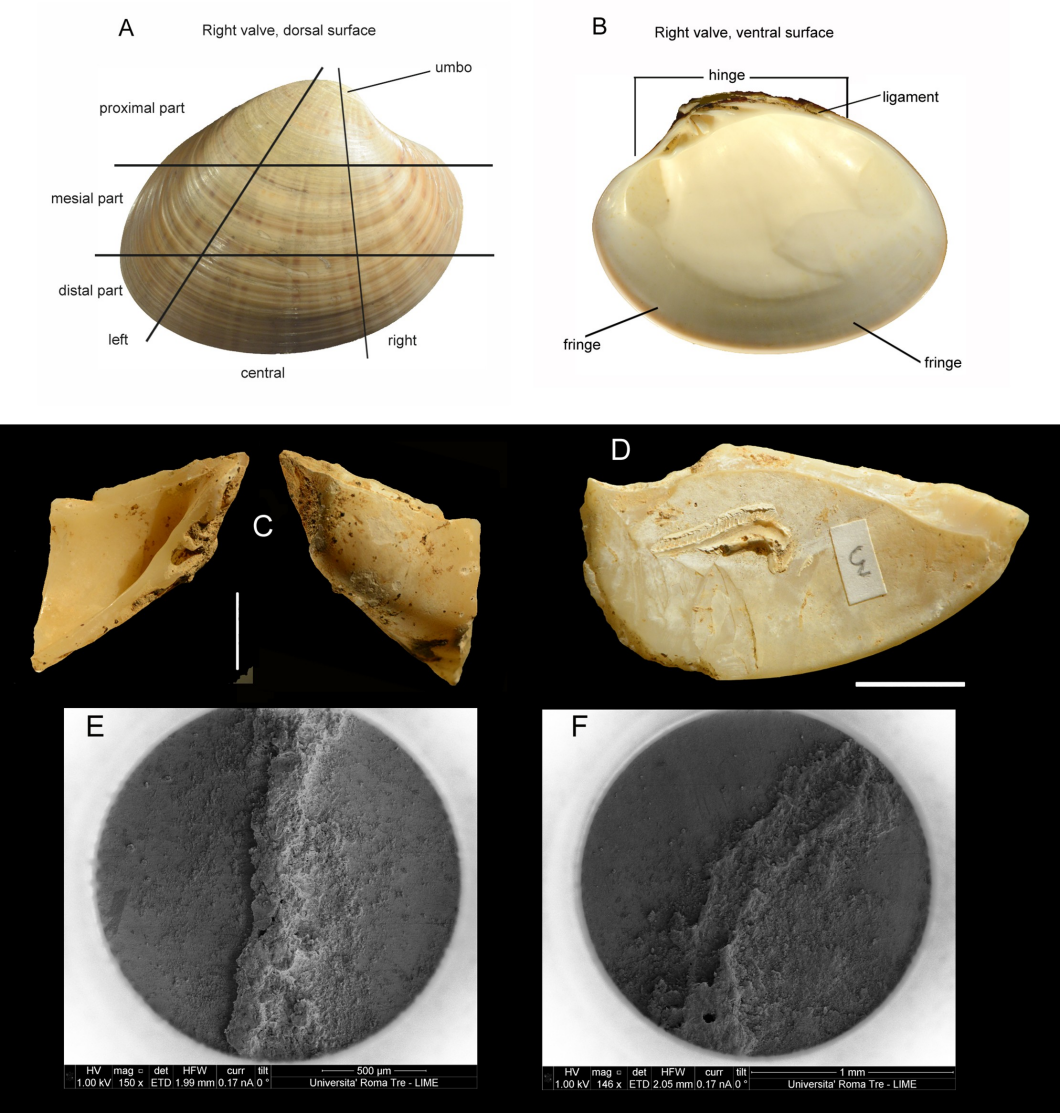
(A, B) Modern left valve of *Callista chione*: (A) external (dorsal) surface; (B) internal (ventral) surface. Glossary of valve parts as in [24]. (C) no. PV7, fragment of a right valve with umbo and hinge, retouch on external face affected by dissolution. This is a beached specimen. (D) no. 3, left valve, retouched fragment of distal fringe. Two slightly abraded serpulid calcareous tubes are encrusted on the internal face. Serpulid worms generally attach themselves to the outer valve; the presence of remnants of the worm protective tube indicates that the bivalve died at sea and the shell was later beached. (E) FIB (Focused Ion Beam) image of a well preserved serpulid tube, on Moscerini no. 185 layer 24, a specimen gathered from the sea floor; (F) FIB image of an abraded serpulid tube, on Moscerini no. 153 layer 21, beached specimen. Both images courtesy of Carlo Smriglio and Andrea di Giulio. The photographs were taken at the LIME (Interdepartmental Laboratory of Electron Microscopy) of Roma Tre University with a Dualbeam FIB/SEM-Helios Nanolab (FEI Company, Eindhoven, The Netherlands). Scale bar of C and D = 1 cm.

Taphonomic characteristics are described as in [31]. Parts of valve (Fig 6A and 6B) morphometric features and temporal relations between retouch and breaks are coded as in [24]. The Moscerini shell tools were, until now, known only from a study by Silvana Vitagliano [4] who inventoried about 100 retouched pieces. The 17 pieces drawn in her publication are those now kept in the Pigorini Museum.

### Procurement of shells

According to [24] the valves of *Callista chione* at Grotta del Cavallo were collected by Neandertals after the death of the mollusk on the nearby beach. During storms shells can be thrown on the beach by waves. Clues used were the presence of abrasion, perforations caused by marine organisms, dissolution and encrustations by barnacles and other aquatic animals on the ventral surface of the shell. However the criteria used to identify beached specimens are not present on all specimens so we were not sure that all shells were routinely collected from the beach as opposed to gathering from the sea floor.

**Fig 7:**
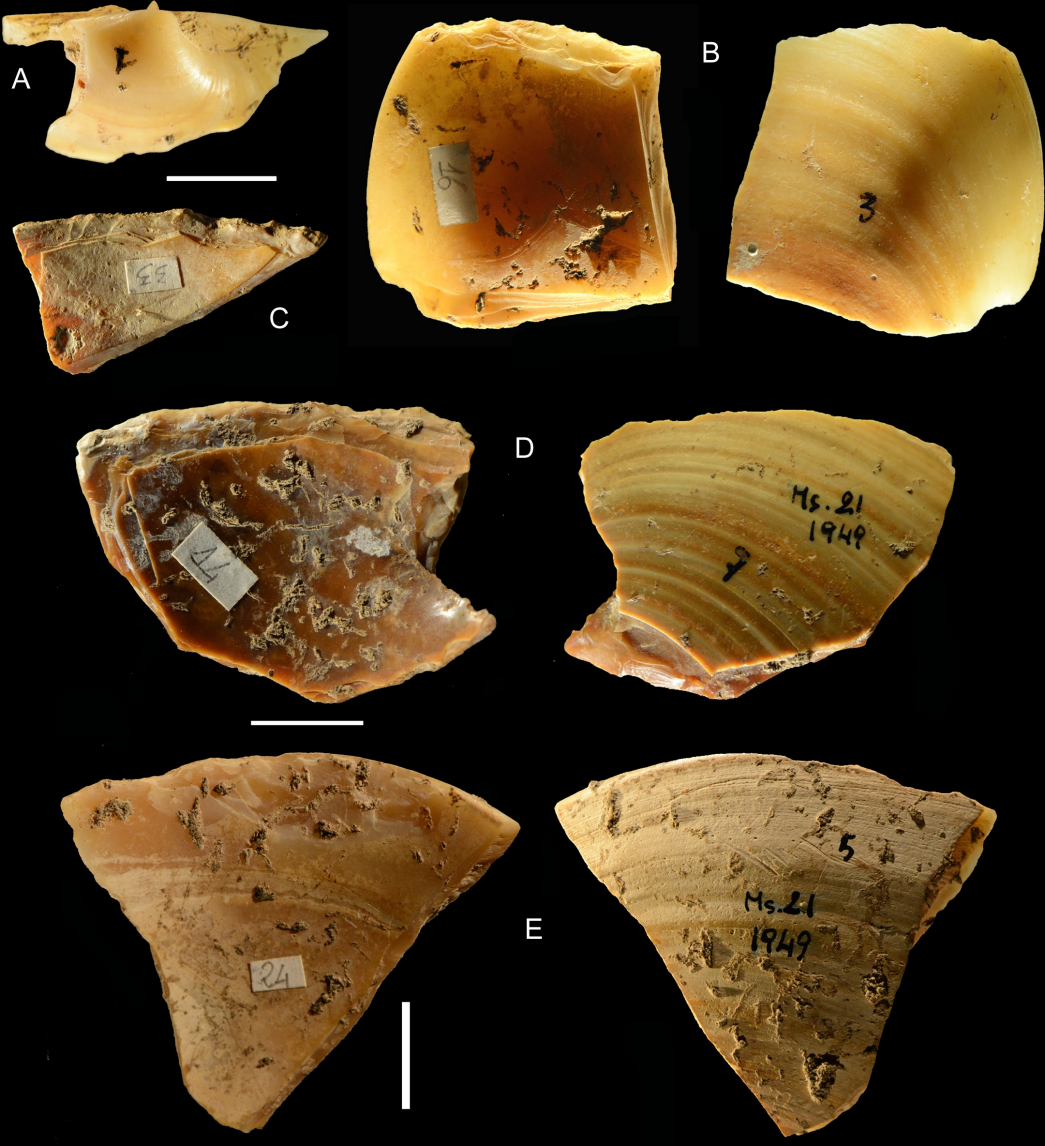
Specimens of *Callista chione* some gathered from the sea floor (A, B, D) and some washed up on the beach (C, E). (A) No.74, unretouched umbo. The unabraded and shiny surface indicates that the bivalve was gathered from the sea floor. (B) No. 16, left valve, the break to the right is posterior to the retouch but the retouched edge is complete; note the shiny dorsal surface. Pits are post-depositional, not borings due to marine organisms because they are present also in retouched areas; (C) No. 33, the retouched edge is broken on the left side, the internal and external surfaces have an opaque, chalky appearance with dissolution indicating that the shell was washed up and collected on the beach. (D) no. 14, mesial part of a right valve covered with postdepositional concretions and a retouched edge broken on the right side but the break is prior to retouch; the unabraded and shiny dorsal surface shows that the shell was gathered from the sea floor. (E) No. 24, left valve with an intact portion of the fringe to the right, the left side is broken but the fracture is prior to the retouch; the eroded band following the growth lines and the opaque external surface indicate a beached specimen. Scale bar = 1 cm.

**Fig 8:**
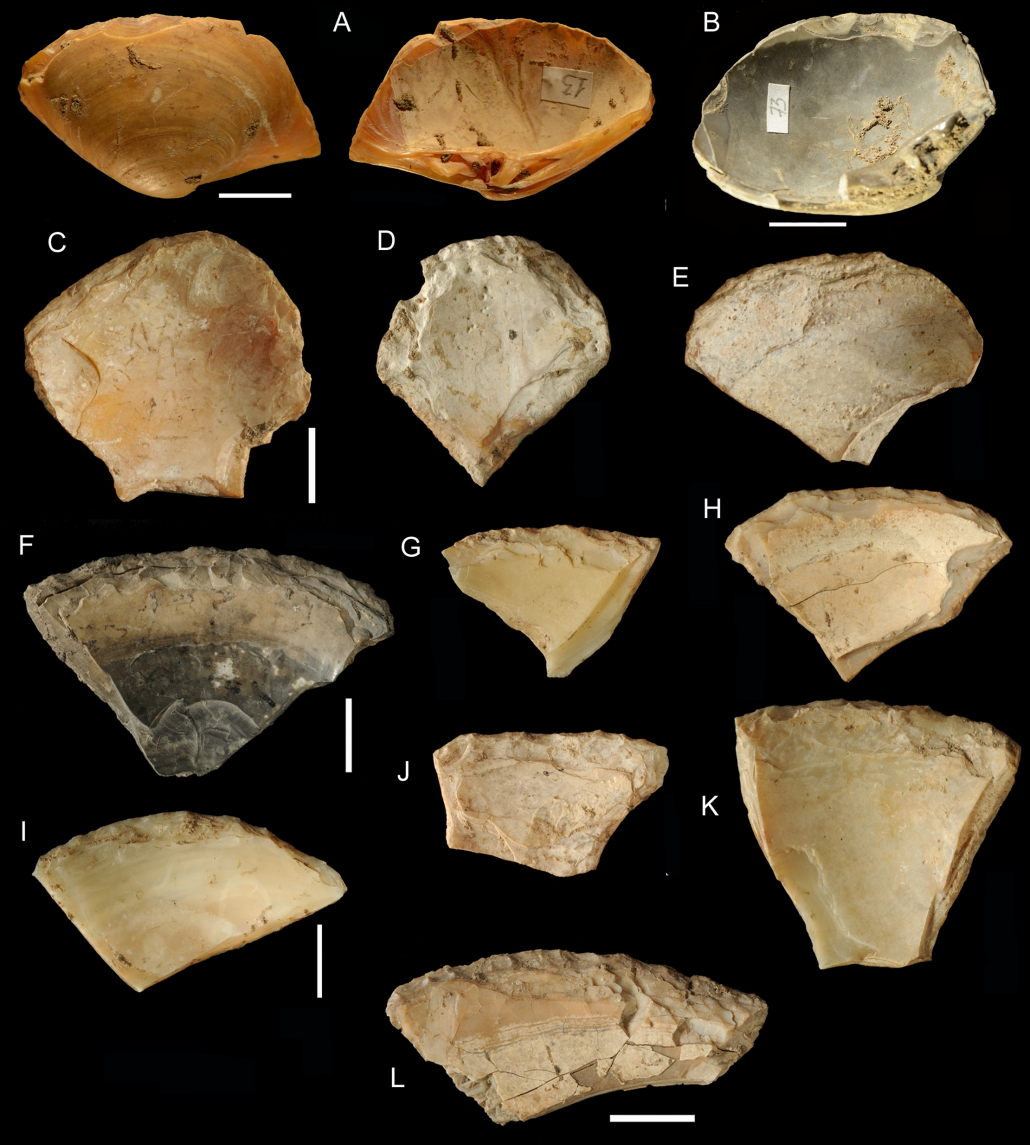
General morphology of retouched shell tools, Figs C-L are from the Pigorini Museum. (A) No. 13, almost complete small shell preserving the umbo and the hinge. The retouched edge is on the dorsal face, a rare occurrence. (B) No.73, almost complete shell preserving the umbo and the hinge; burnt after retouch. (C) No. 106182, the retouched edge is complete. (D) No. 106190 with three complete retouched edges. (E) No. 106192 the edge is complete. (F) No. 106183 the retouched edge is complete, burnt after retouch. (G, H, I, K) Nos. 106189, 106184, 106185, 106185 the retouched edge is complete. (J, L) Nos. 106188, 106195 the breaks at the base do not affect the retouch. Scale bar = 1 cm.

One of us, Carlo Smriglio who is a specialist of Mediterranean modern and fossil shells, examined under a microscope all the Moscerini specimens in the Italian Institute of Human Paleontology. Beached specimens can be distinguished from specimens that were collected as live animals in the sea, using a number of criteria: (a) degree of opacity or shine of the outer valve; (b) state of preservation of the internal and external surfaces; (c) presence or absence of encrusting marine organisms; (d) traces of abrasion or rounding of the hinge (Figs 6–8). Our data base indicates that 23.9% of the specimens (40 of 167 that could be determined) were gathered from the sea floor (Table 4**)**.

**Table 4:** Grotta dei Moscerini, procurement of retouched shells (undetermined cases excluded).

Level	Beached	Gathered from sea floor	Total
14	1	0	1
17	1	0	1
18	3	0	3
20	2	0	2
21	24	2	26
22	26	2	28
23	9	7	16
24	13	10	23
25	10	8	18
26	17	4	21
35	2	0	2
36	1	0	1
37	7	1	8
38	4	3	7
39	6	3	9
42	1	0	1
**Total**	**127**	**40**	**167**

The specimens stored in the Pigorini Museum were excluded from the analysis because they are covered by preservative. Four specimens clearly gathered from the sea floor were included in that category although they were too broken for determination of retouch.

In modern times the clam is fished by dredging, that is, using small boats towing dredges over the sea bottom. Dredges consist of a small (45 to 70 cm) semicircular iron bar with an attached net bag or metallic grid and three or six metal rods that scoop the sandy bottom [32]. It is also collected by hand by scuba divers working in shallow coastal waters (<10 m) along the Adriatic coast of Croatia [33]. In the northern part of the Adriatic Sea there are sand banks where *Callista* can be collected at < 0.5–1 m depth [34].

### Skin diving by Neandertals

Gathering of the shells from the sea floor requires wading or most often diving in shallow water. Clams can be close to the shore and their syphon makes them visible under water. They can be gathered by hand scooping the sandy bottom. Gathering by hand without breathing equipment, as Neandertals must have done, clearly cannot be as effective as dredging or scuba diving. Frequencies of *Callista chione* in the Moscerini layers are never very high, although at that time, in the absence of commercial exploitation, *Callista* populations were probably more numerous than now.

Bony growth in the external ear canal is correlated with habitual exposure to cold water [35]. These bony growths (exostoses) are quite common on Neanderthal remains and the most likely explanation is that Neanderthals spent a good deal of time collecting aquatic resources [35; see also 36–37]. The archaeological evidence from Moscerini supports the idea of frequent aquatic resource exploitation based on anatomical data.

Fishing in shallow waters is also documented in the Mousterian of Castelcivita cave (Southern Italy) by remains of freshwater fish (trout, chub and eel, NISP = 21) [38]. Additional evidence of freshwater fish exploitation is provided by some French sites (Payre, Abri du Maras) [39–40], Kudaro cave in the Caucasus [41] and Spanish sites (Vanguard and Gorham caves in Gibraltar, Bajondillo, Abrigo 3 and Nerja cave on the coast of Malaga). The most common resource in Spanish costal sites is the mussel [42–44].

Fishing in shallow fresh water fish or collecting shells from rocky shores or coastal sandy sea floor by skin diving is a simple technology. There is an increase in aquatic resource exploitation in the Upper Paleolithic (UP thereafter) but a decrease in the frequency of exostoses in the external auditory canal on the UP fossils [35]. This is possibly related to the use of more sophisticated fishing technologies such as fishhooks or barbed spears, which do not require diving. Double-pointed fishhooks are documented in the Châtelperronian and the Aurignacian, the curved ones are abundant in the Magdalenian [45]. We have no evidence for nets in the UP but twisted fibers possibly used for cordage are documented at Abri du Maras in a layer dated to MIS4 [40]. Other evidence of cordage with three twisted strands of vegetal fibers is from the cave of Lascaux [46] and evidence of fiber twisting in the UP of Pavlov (Czech Republic) [47].

Contrary to the idea that Neandertals were incapable of systematic use of coastal resource [48], the lower intensity of shell fishing in the Mediterranean compared to South African Middle Stone Age sites is due to the fact that the Mediterranean coasts support a lower mollusk biomass than oceanic littorals for a number of reasons. It lacks significant upwelling by which wind-displaced surface waters are replaced by cold, nutrient-rich water; the intertidal areas on the coasts of South Africa supported a good number of large mollusks (limpets, mussels) and during spring low tides their maximal exposure allowed intensive shell fishing collection. But tidal oscillations in the Mediterranean are generally of the order of few cm while on the south coast of South Africa the difference between low tide and high tide is about 1 m. [26, 49].

#### Consumption of *Callista chione*?

Placing bivalves on the hot embers of a fireplace will cause opening of the valves, allowing the consumption of the mollusk [50] and causing darkening of the shell. As discussed by [2] the fact that some shells are burned (22.2% of the retouched pieces; 35% of Callista hinges [2: table 6.13]) might suggest cooking and alimentary consumption of the mollusk prior to retouching of the shell but this is not necessarily so because 37.5% of the stone artifacts are also burned and many layers from 43 to 11 contain ashes and charcoal lenses. Moreover some shell pieces were burned after retouching so the hypothesis of cooking is not supported (Fig 7B and 7F; Fig 8A and 8C).

In conclusion, *Callista* clams were apparently collected as part of a routine of getting raw materials for tools (see section “Relationship of stone and shell tools”). Nevertheless marine bivalves can easily be opened and eaten raw [50] so the hypothesis of consumption of at least some of *Callista chione* cannot be excluded.

#### Blanks of shell tools

Only two specimens are made on an almost complete shell retaining the umbo and hinge (Fig 8A and 8B) and 11 are on fragments with only the hinge and the umbo (Fig 6C). All other retouched shells are on fragments without umbo and hinge (Table 5). According to Stiner [2: table 6.13] the total number of is 182. The retouched specimens are 13 and the unretouched specimens are 169 (182–13 = 169) which represent 93% of the total (169/182). This means that hinge and umbo were removed, rarely used as blanks and were set aside as waste products. A comparable number of unretouched umbos and hinges occur at Grotta del Cavallo where retouched specimens are 5 on a total of 82 [24]. Thus the unretouched umbos and hinges at Cavallo are 94% (77/82), very much like at Moscerini.

**Table 5:** Grotta dei Moscerini, blanks of retouched shells. Forty six cases on an undetermined part are excluded.

Part	N	%
Almost complete shell	2	1.6
Umbo and Hinge	11	8.8
Proximal	19	15.2
Mesial	59	47.2
Distal	34	27.2
Total	125	100

“Almost complete” means a whole or only minimally fragmented shell that retains umbo and hinge. Only two specimens fall in this category (Fig 8A and 8B). The “proximal” part may include small portions of the mesial and distal parts but does not include the umbo and the hinge. In other words, most of the retouched *Callista* are fragments without the umbo and hinge. The very large number of unretouched umbos and hinges suggests that knapping of a shell to make it into a tool started by removal of the umbo which is the most prominent part of the shell with maximum curvature of the dorsal face, to obtain fragments that resemble most the flat or slightly curved profile of a stone flake.

Romagnoli et al. [24] suggest that retouching was initially done on the whole or only partially fragmented shell and that fragments were most often the result of voluntary impact [24 figs 7,11; 25, p. 225] However experimental work by Sylvain Soriano shows that knapping often causes fragmentation of the shell in two or more fragments. Thus the high proportion of fragmented blanks in the assemblage may be the result of the brittleness and inhomogeneity of the shell texture and its reaction to knapping, though break through use may also have been a factor. More details on knapping methods are provided in S5 File.

There are 25 retouched shells that are either complete (Fig 8A-8I and 8K) or the breakage does not affect the retouched edge (Fig 8J and 8L). But the great majority of retouched shells (= 146) have fractures that affect one or two retouched sides (Fig 7C and 7D).

#### Morphology

Information on the desired shape of shell tools is provided by the temporal relations of retouch and breaks, a variable discussed by [24] who suggest that retouching was initiated on the proximal fringe (Fig 6). In fact the variable can be used to reveal the desired shape of shell tools. The completeness of retouch scars can be evaluated in the same way that lithic analysts do to establish the sequence of knapping scars, using features such as lateral symmetry of the removal, its orientation and direction, and the intersection between the scar edge and the break surface.

**Fig 9:**
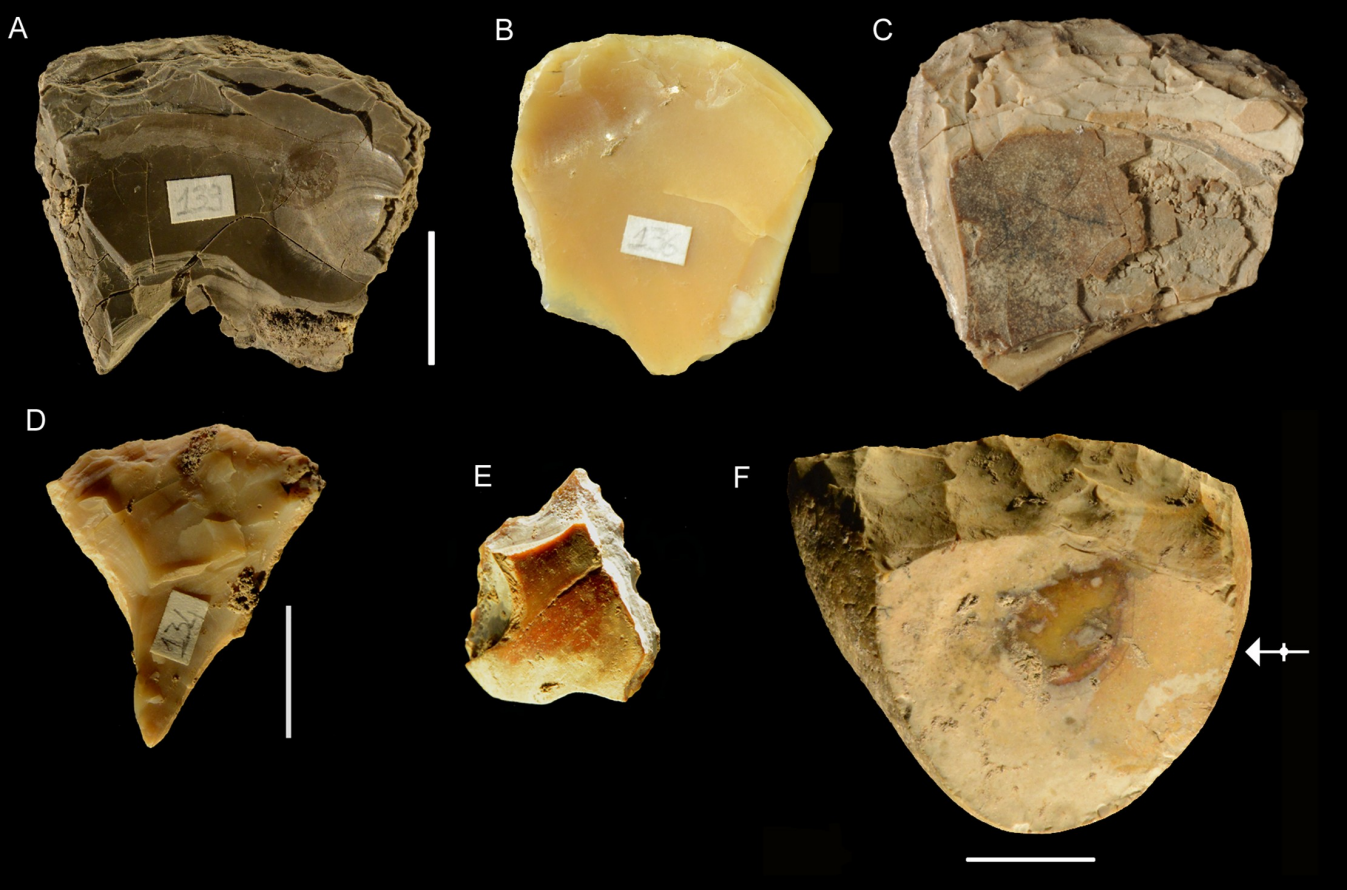
(A and C) Nos. 139 and 106191 burnt after retouching. (B) No. 136, left valve with retouch on distal fringe, the left side is broken. (D) No. 134, the retouched edge is intact; the lateral breaks precede the retouch. (E). No. 20, a very small denticulate, 17 mm long with retouch on the external face. This is one of a kind. (F) No. 106014, flint scraper from level 14, with an edge comparable to the fan-shaped edges of retouched shells. Note however that this is really a side scraper on a flake; the symbol indicates the position of a cortical butt and the debitage direction. Scale bar = 1 cm.

Aside from two almost complete shells (Fig 8A and 8B) there are 21 pieces which are on shell fragments and where the retouch on one or two edges is superimposed on one or two previous breaks and is complete (Fig 8C-8I and 8K; Fig 9D and 9E). Two more pieces have a break at the base but the retouched edge is complete (Fig 8J and 8L). Most pieces have just one retouched edge (78 of 94; broken pieces excluded); pieces with more than one retouched edge are rare (Fig 8D and Fig 9E).The fan-shaped edge of these pieces seems dependent on the general shell morphology; it is almost inexistent among the stone artifacts (Fig 9F). All other pieces have at least one retouched edge affected by a break, but the great majority is affected by more than one break. We do not know if these shells were hafted to a wooden haft using a leather binding or a vegetal string. The removal of umbos and the trapezoidal or triangular shape of the shell tools may be related to hafting but we have no proof of it.

Differently from Grotta del Cavallo, retouch at Moscerini was not always done on the internal (ventral) surface of the valve, a small number of elements have retouch on the external face or alternating (Table 6; Fig 6C, Fig 8A). The laterality of the valves is provided in Table 2 in S1 File).

**Table 6:** Position of retouch. Indeterminate cases are excluded.

Position of retouch	N	%
Internal surface	140	87
External surface	13	8.1
Both on internal and external surface	8	5
Total	161	100.0

#### Retouched edge

In most of Italian MP sites where retouched seashells have been described *Callista chione* was preferably or exclusively selected. Shells of *C*. *chione* may have been preferred for their sturdiness, their morphology and valve size. Unlike some other species from the Veneridae family, *C*. *chione* is free from periostracal calcification [(i.e. needles and pins); [51])] so the outer surface of the shell is smooth. Thus the retouched valve retains a regular edge. As demonstrated by studies relative to dredging damages, *C*. *chione* has a very strong and resistant shell compared to other species [29].

Preliminary microwear analysis of *C*. *chione* retouched shells from layer L at Grotta del Cavallo concluded that the distribution of striations, which are of longitudinal orientation in seven cases and transversal in only one case, allows the identification of the action done but the identification of the worked material was not possible [52]. SEM analysis of the Moscerini retouched shells at the Pigorini Museum done at the request by Emanuela Cristiani has shown that they were covered by preservative and could not be studied for microwear.

Gathering shells from the sea floor is more time-consuming than collecting specimens on the beach. However shells gathered from the sea floor tend to be thicker than beached specimens and this may have been a reason for choosing them. The average thickness (measured on the valve just below the retouched edge) of beached shells is 2.5 mm ± 07. The average thickness of shells from the sea floor is 2.7 mm ± 0.5. However both kinds of populations contain specimens with a heavy shell and others with a light shell so the short duration of the occupation may have favored beach shells.

### Shell tools in time and space

The oldest evidence of use of shells as tools is documented from Trinil (Java) in the same deposit that yielded the skull and femur of *Homo erectus* [53]. An assemblage of 190 whole and fragments of freshwater mussel shells (*Pseudodon vondembuschianus*) representing a minimum number of 166 individuals, excavated in 1890, and dated between 0.54 and 0.43 million years, were collected, opened and presumably eaten by *Homo erectus*. One shell had a geometric pattern incised on its surface. Only one other shell had retouch on its ventral margin. A hypothesis of use by *Homo erectus* of unretouched fragments of clam shells for butchering had previously been suggested based on older finds of cutmarked bovid bones from Sangiran [(central Java); 54].

Unretouched limpet shells (*Patella* sp. and *Patella vulgata*) were used at the cave site of Altamira where human occupation layers at the cave entrance are dated from the Gravettian to the Magdalenian, between 26 and 17 ka cal BP. Use-wear analysis showed that 57 limpets out of 602 specimens, primarily collected for consumption, were then used for scraping ochre utilized to paint the graphic representations in the cave [55]. Use of shell to obtain pigment is also documented at two other Upper Paleolithic Spanish cave sites (Fuente del Salin and Tito Bustillo in Cantabria) [56]. At Fuente del Salin two fragments of *Patella* were retouched to make them into borers [57].

Use of marine mussels (*Mytilus galloprovincialis*) as tools is documented at a much later period, in eight caves and open-air sites dated to the Late Mesolithic and Neolithic of Southern France, most of them quite far away from the coast. Striations and polish on the unretouched mussel edges with red pigment residues (hematite and bauxite) suggest that the shells were used for scraping and cleaning animal skin and perhaps finishing ceramic vases [58].

A small number (n = 7) of retouched opercula from the *Turbo marmoratus*, a large marine gastropod have been found in the Golo cave, located in the Gebe Island, eastern Indonesia [59]. *Turbo marmoratus* knapped opercula were associated with a small lithic assemblage (n = 51) in a deposit dated between 32,000 and 28,000 BP. The operculum is a circular, smooth and thick calcareous feature that serves to seal the entrance of the shell. The knapped pieces illustrated in [59: fig 4] have a diameter of about 7–8 cm They are knapped by continuous unifacial retouch along its outer edge to form a scraper-like tool with a convex semi-abrupt working edge. *Turbo marmoratus* lives in crevices on reef crests and gathering of the shell probably required shallow diving.

In sum, there are examples of unretouched shell tools and shells retouched as stone artifacts in various times and in different regions. At present however only some Italian assemblages and one in Greece show a systematic use and retouching of shells as tools. The following list of Mousterian sites with retouched shells (Table 7) is based on papers by [23,24], excavation reports and accounts by cited authors. In all Mousterian sites in Italy and Greece shell tools were made on *Callista chione* [24]. Worked examples of *Glycymeris* are cited for two caves (Uluzzo C and Mario Bernardini) but the identification needs to be confirmed (23).

**Table 7:** Retouched shell tools in Mousterian sites.

Site	Region	Retouched *Callista chione*	Age	Reference
Ex-Casinó-Balzi Rossi	Liguria, Italy	About 40	undated	[23, 60–61]
Grotta dei Moscerini	Latium, Italy	171	Mis 5 to MIS 4	[4] this paper
Grotta del Cavallo	Apulia, Italy	126, well-documented	MIS 5e-MIS 5d	[24, 62]
Grotta Mario Bernardini	Apulia, Italy	45 fragments but badly preserved and undescribed	undated	[23]
Grotta di Serra Cicora	Apulia, Italy	Possibly 13 but only one is illustrated	undated	[23]
Grotta di Uluzzo C	Apulia, Italy	3	undated	[23, 63]
Grotta di Torre dell’Alto	Apulia, Italy	One retouched and 3 unretouched fragments	undated	[23, 64]
Grotta Marcello Zei	Apulia, Italy	96 fragments, only 2 are retouched	undated	[23, 65]
Grotta di Capelvenere	Apulia, Italy	A few, no description available	undated	[23]
Grotta dei Giganti	Apulia, Italy	18 shell fragments, 8 are retouched	undated	[23]
Kalamakia	Peloponnese, Greece	At least 6 pieces	undated	[23, 66]

Two other occurrences at Riparo Mochi and Barma Grande, with one retouched piece each, are mentioned [61] but remain undocumented and are excluded from this table.

### Pumices

**Fig 10:**
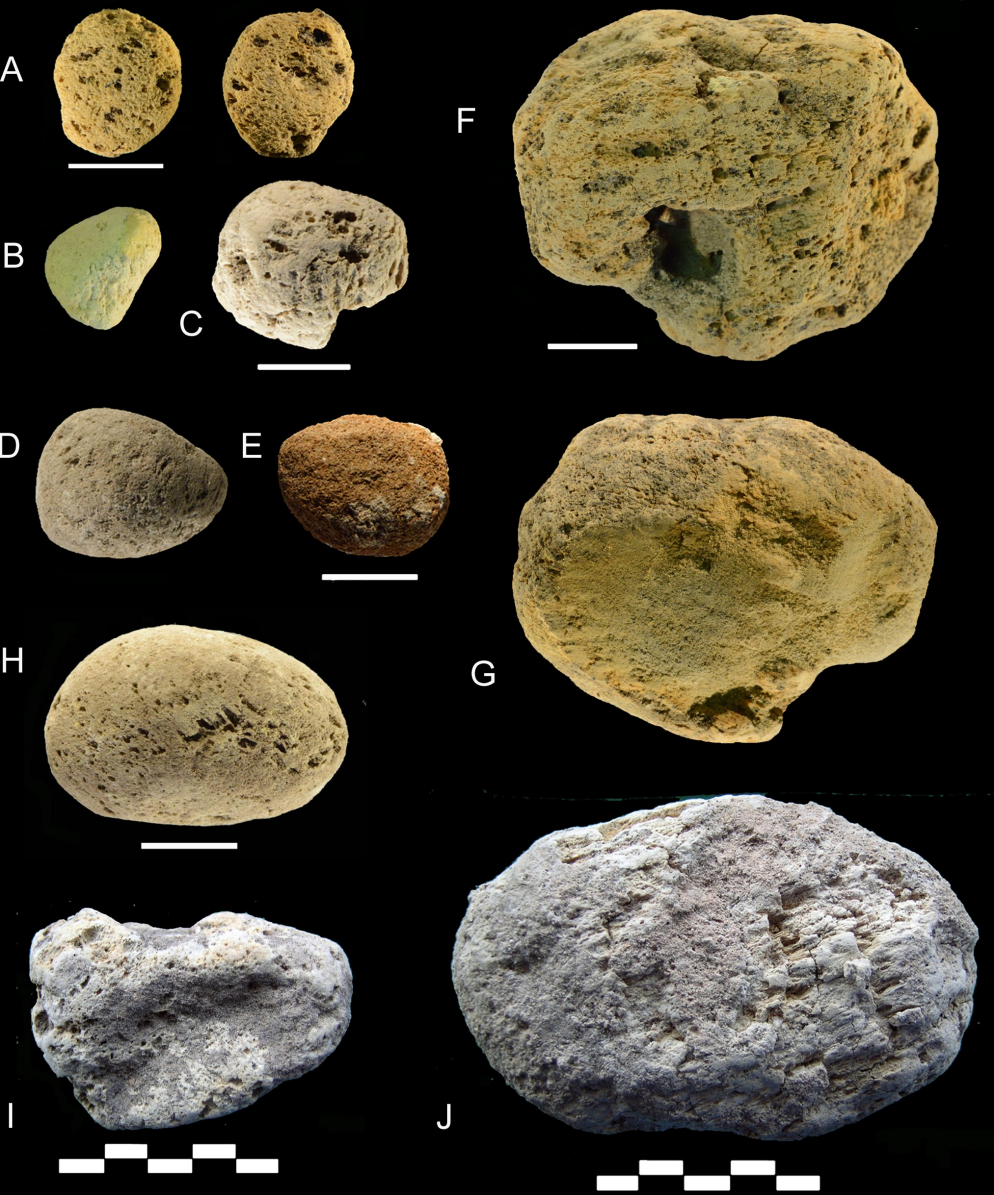
Pumices from layers 19, 20, 21, 22, 37. (A) layer 19 no. 1; (B) layer 20 no. 6; (C) layer 21 no. 19; (D,E,H,I) layer 22 nos. 37,38,26, and no number; (F-G) layer 20 no. 5; (J) layer 37 no number. Scale of A-H = 1 cm; scale of I, J = 5 cm.

**Fig 11:**
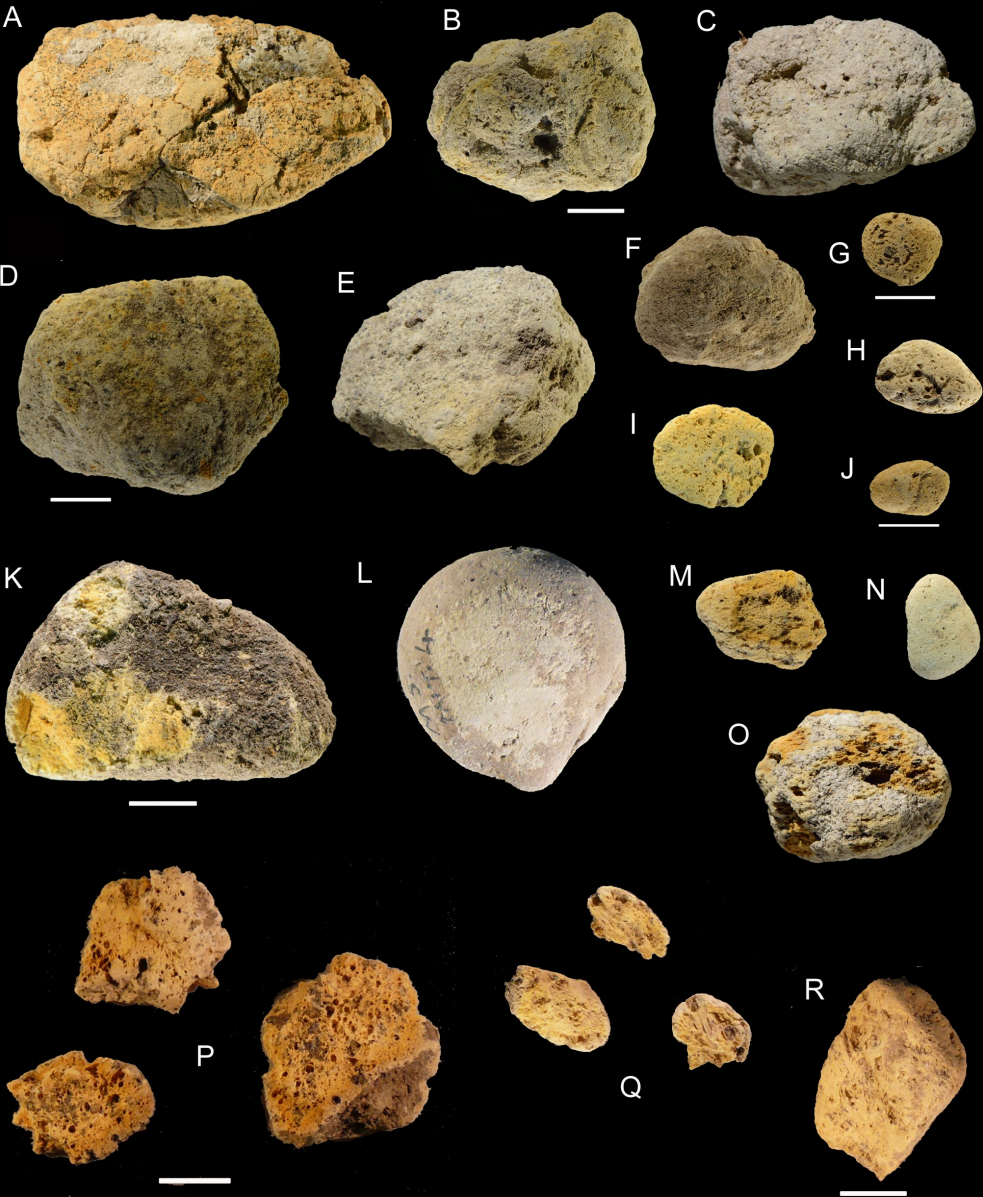
Whole pumices from layers Interno 1c, 21, 22, 23, 24, 25, 30 and fragments from layer 21, 29 and Interno 3. (A) Interno 1c, no number; (B) layer 21 no. 16; (C) layer 21 no. 18; (D) layer 21 no. 17; (E) layer 22 no. 25; (F) layer 22 no. 27; (G) layer 22 no. 39; (H) layer 23 no. 11; (I) layer 23 no. 8; (J) layer 23 no. 12; (K) layer 25 no. 4; (L) layer Interno 4A; (M) layer 23 no. 13; (N) layer 30 no. 7; (O) layer 24 no. 21; (P) three fragments from layer 21; (Q) three fragments from Interno 3; (R) fragment from layer 29. Scale = 1 cm.

Several layers of Moscerini cave contain one or more pumices (Table 8; Figs 10 and 11). This is an unexpected occurrence, to our knowledge never mentioned in papers on Neandertal sites, with a single exception (see section “Analysis of Santa Lucia pumice”).

**Table 8:** Grotta dei Moscerini. Counts of pumices by layers.

Layers	No. pieces	Catalogue number	Length (maximum dimension) in mm (in sequence as per catalogue number). Complete pieces only.
16		rare fragments of yellow pumices, mentioned by Segre, not seen by us	
19	1	1	16
20	2	5,6	50, 11
21	7	16,17,18,19, 20, three fragments taken by Marra	40, 44, 42, 21, 26
22	16	25,26,27,37,38,39 nine small fragments (not in photos) nos. 28–36. No. 38 given to F. Marra. One piece without number taken by Marra.	43,31,29,20,18,13, no number 60. Plus 9 fragments 10–28 mm in length.
23	8	8,9,10,11,12,13, two small fragments nos 14,15	20, 21, 18, 18, 13, 19
24	4	21,24 (22,23 fragments, no photos)	32, 34
25	1	4	45
26	1	2	31
29	1	taken by F. Marra, yellow pumice mentioned by Segre	
30	1	7	15
35–36		white pumices mentioned by Segre, not seen by us	
37	1	taken by F. Marra	100
Interno 1c	1	3, given to Marra	68
Interno 3	3	taken by F. Marra	
Interno 4	2	40 and 1 fragment, taken by F. Marra	4.1
44	many	pumices mentioned by Segre, not seen by us	

We catalogued, measured and took photos of 49 pumices or pumice fragments, stored in the IsIPU storage area in the town of Anagni (Latium, Italy). Fabrizio Marra (Istituto Nazionale di Geofisica e Vulcanologia) took 11 pieces for thin sections and geological analysis by Mario Gaeta (University of Rome La Sapienza) and dating by Brian Jicha (University of Wisconsin). Marra’s samples did not receive a catalogue number, except for two first catalogued by us (no. 38 level 22 and no. 3 Interno 1C). Only complete pieces have been measured.

The average size (maximum length) of pumices that could be measured varies from 1 to 10 cm, with an average size of 3.0 cm. These pumices are fragile and porous, often fragmented.

### Origin of Moscerini cave pumices

Five pumices from the following layers were analyzed

- T4 from Interno 4 (Fig 11L)
- T3 from Interno 3 (Fig 11Q)
- 37 from layer 37 (Fig 10J)
- 22 from layer 22 (Fig 10E)
- 21 from layer 21 (Fig 11P)

The analytical techniques are provided in the Supporting Information (S1 File).

#### Texture and glass composition of Moscerini cave pumices

The five pumices analyzed in thin sections are vesicular and poorly porphyritic, with two samples (T4 and 21) practically aphiric and three samples (37, 22 and T3) showing a total phenocryst content up to 2 vol%. Feldspars are the dominant phenocrysts occurring, generally, as glomerocrysts (Fig 2a in S1 File). Additional phenocryst phases include black mica and clinopyroxene (in decreasing order of abundance): opaque oxide minerals, apatite and sphene are accessory phases. The porphyritic pumices show a variable mineral assemblages. Pumice 37 is characterized by dominant alkali-feldspar, followed by biotite (Fig 2b in S1 File) and accessory phases; the groundmass is mostly glassy with rare microlites that are not optically resolvable. Pumice 22 is made up of sanidine phenocrysts in a glassy groundmass. Pumice T3 is constituted by dominant plagioclase, minor amounts of black mica, rare clinopyroxene and accessory phases.

**Fig 12:**
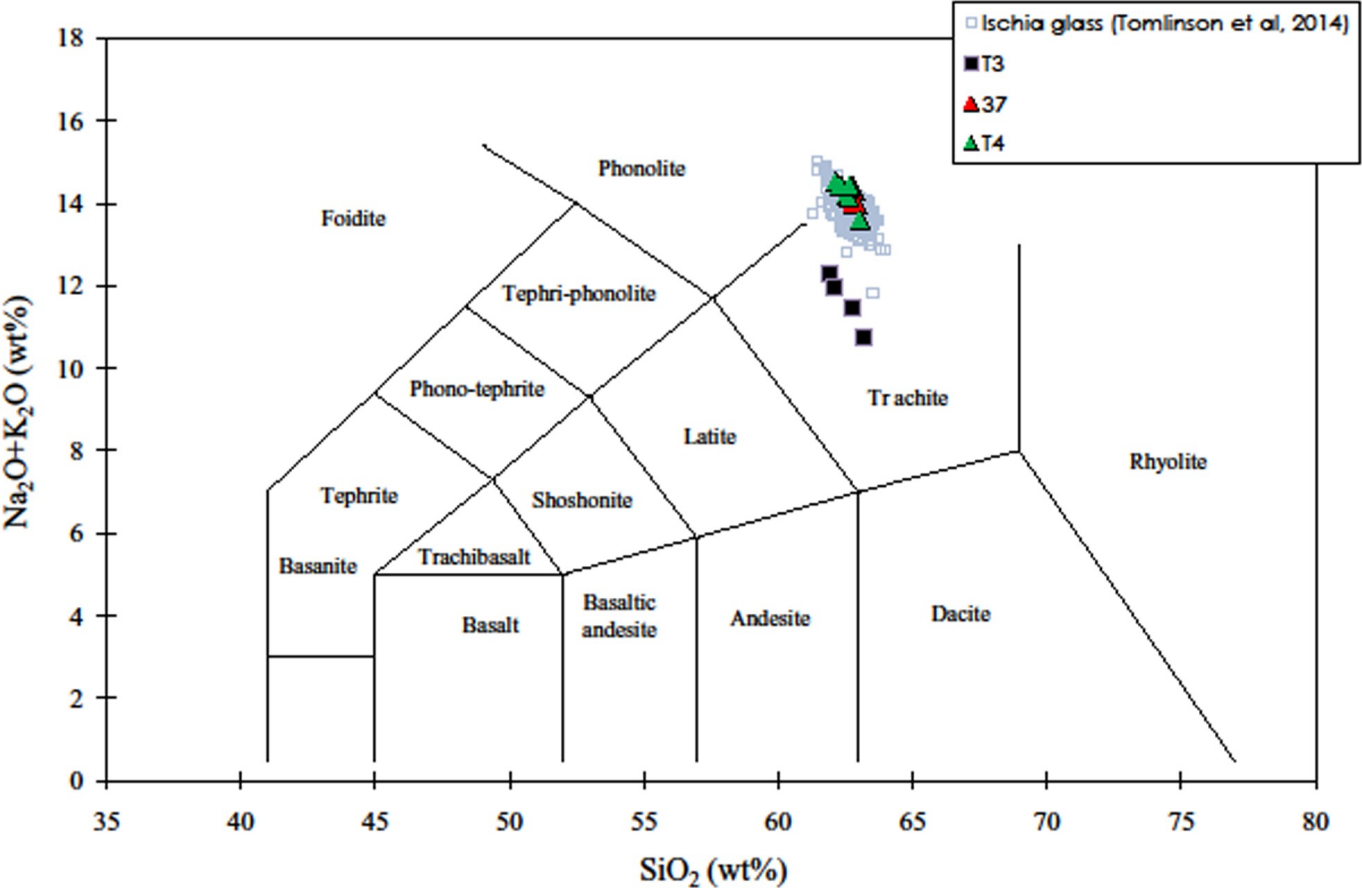
**Total alkali (Na**_**2**_**O + K**_**2**_**O) *vs* silica (SiO**_**2**_**) diagram [(TAS), 67] illustrating the composition of glasses in the Moscerini cave pumice, recalculated on an anhydrous basis.** Data of glasses in the Ischia pumice are reported for comparison.

Also in this sample the groundmass is mostly glassy with rare microlites that are not optically resolvable. Pumice 37 and T4 have an alkali-rich composition at the border between the phonolitic and trachytic field, while pumice T3, displays trachytic composition in the TAS diagram (Fig 12).

The content of Cl above 0.40 wt% (Table 9) indicate that the analyzed pumices belong to the Campanian volcanoes (i.e. Campi Flegrei, Vesuvio and Ischia) [68]. In particular, the K_2_O/Na_2_O and CaO/FeO ratios (Table 9) and the mineralogical assemblage of pumice 37 and T4 match those of Tufo Verde Epomeo (TVE) and post-TVE pumice of Ischia whose age is bracketed in the interval ~75–50 ka [69]. The trachytic pumice T3 display a composition that does not match those of Ischia products. According to [68] the higher CaO/FeO ratio (Table 9) of T3 pumice is typical of trachytes erupted by Campi Flegrei. Tentatively, the composition of T3 pumice is comparable those of pumice erupted before the Campanian Ignimbrite [70]. This eruptive period is poorly known from a geochronologic point of view; however, regional tephrostratigraphic data constraint it between 110 and 40 ka [68].

**Table 9:** Averaged glass analysis obtained by electron microprobe.

Sample	37	T4		T3	
	(2)	(7)	sd	(4)	sd
SiO_2_	61.26	59.90	*2*.*16*	61.41	*0*.*80*
TiO_2_	0.62	0.59	*0*.*04*	0.37	*0*.*05*
Al_2_O_3_	17.43	17.20	*0*.*60*	18.59	*0*.*27*
MgO	0.29	0.34	*0*.*04*	0.57	*0*.*03*
CaO	0.96	0.93	*0*.*04*	2.50	*0*.*06*
MnO	0.32	0.36	*0*.*04*	0.16	*0*.*02*
FeO	2.78	2.78	*0*.*14*	3.14	*0*.*10*
Na_2_O	7.87	7.61	*0*.*44*	2.86	*0*.*43*
K_2_O	5.96	5.99	*0*.*27*	8.55	*0*.*25*
P_2_O_5_	0.05	0.03	*0*.*03*	0.11	*0*.*04*
SO_3_	0.07	0.04	*0*.*03*	0.12	*0*.*01*
F	0.38	0.40	*0*.*15*	0.15	*0*.*10*
Cl	0.72	0.76	*0*.*05*	0.45	*0*.*03*
Total:	98.70	96.94		98.97	
K_2_O/Na_2_O	0.8	0.79		3.03	
CaO/FeO	0.3	0.3		0.8	

Number of analyses in brackets; sd = standard deviation.

#### Transport of pumices

The rounded edges of all complete pumices indicate that they had been transported by sea from the source volcano, whether the island of Ischia in the gulf of Naples or from the Campi Flegrei west of Naples. Due to their low specific gravity (in the range of 0.5–0.7 gm/cm^3^) pumices can float and can be transported by marine currents over large distances [71]. Pumices were thus transported to the beach at the base of the Moscerini sequence (layer 44) where they are just flotation deposits.

But how were they then transported in the various layers of the archaeological sequence which is 8 m thick?

Three possible explanations can be suggested for the presence of pumices in the Moscerini sediments. It could be argued that they were transported by the sea that entered the cave in episodes of rise of sea level during MIS 5.The lithodome holes and the wave-cut notch on the north wall of Moscerini cave (Fig 2B) indicate that the sea entered the cave but we believe it must have been prior to the deposits forming the stratigraphic sequence [2: p. 47, based on Segre]. If the sea entered the cave after part of the sequence had been deposited, the layers which have numerous fireplaces, unrolled stone and shell artifacts would have not been preserved. Low-energy waters might leave intact the most internal parts of the sediments but the wave-cut notch indicates high-energy waves, incompatible with the preservation of intact deposits, as studies of submerged caves show [72].

In fact the strongest evidence against sea-water entering the cave, after some or most of the layers forming the stratigraphic sequence had been deposited, is the presence of stalagmitic lenses in many levels, including those with pumices. Fig 3 shows that stalagmitic lenses were noted by A. Segre in 17 Moscerini levels from 1 to 41. Speleothems only grow subaerially, that is in caves that are above sea-level. Thus either the pumices were brought into the cave deposits by gusts of wind picking up shells from the beach at the base of the sequence or by Neandertals who collected pumices.

**Fig 13:**
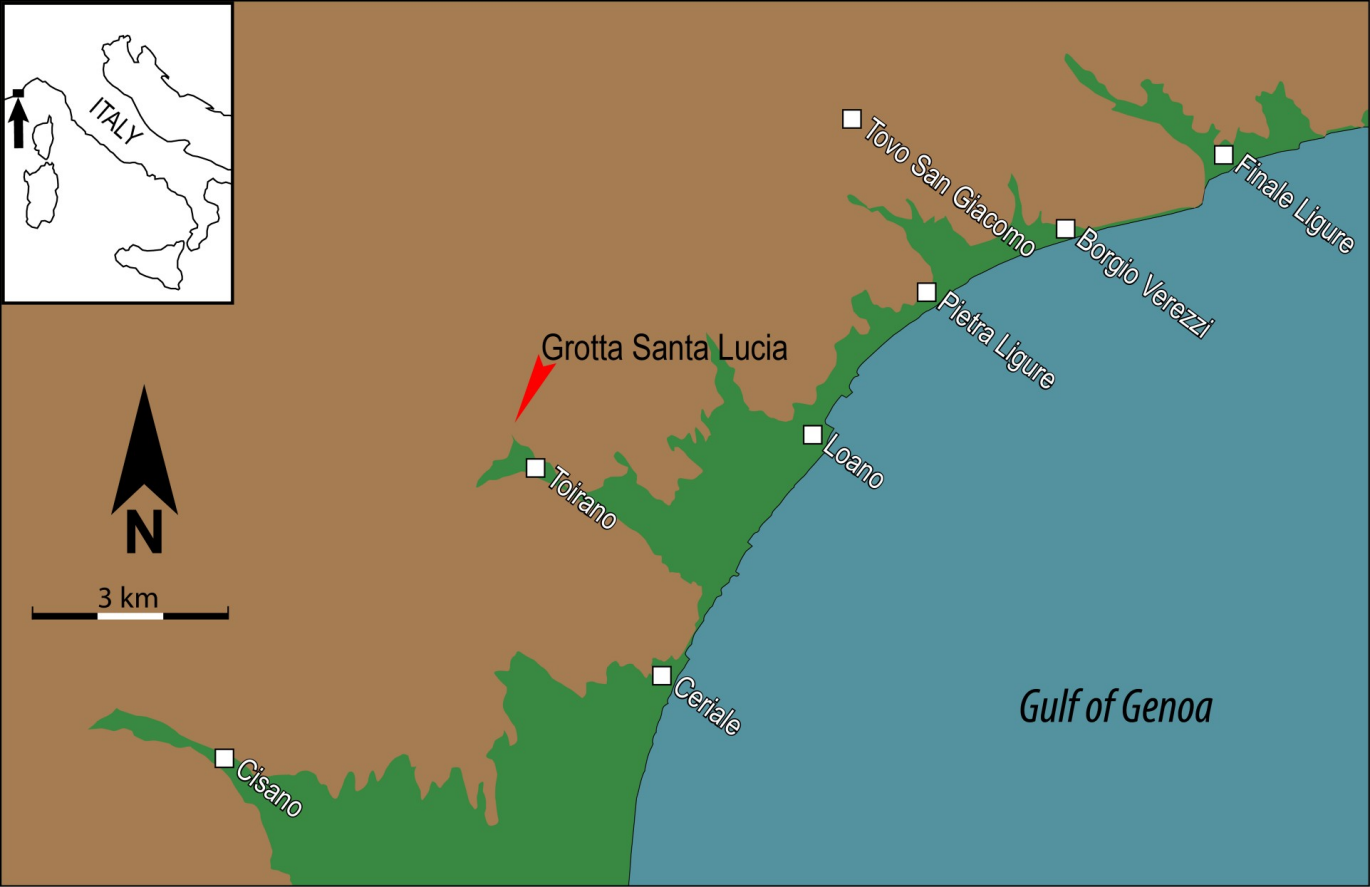
Map of location of the Santa Lucia cave.

**Fig 14:**
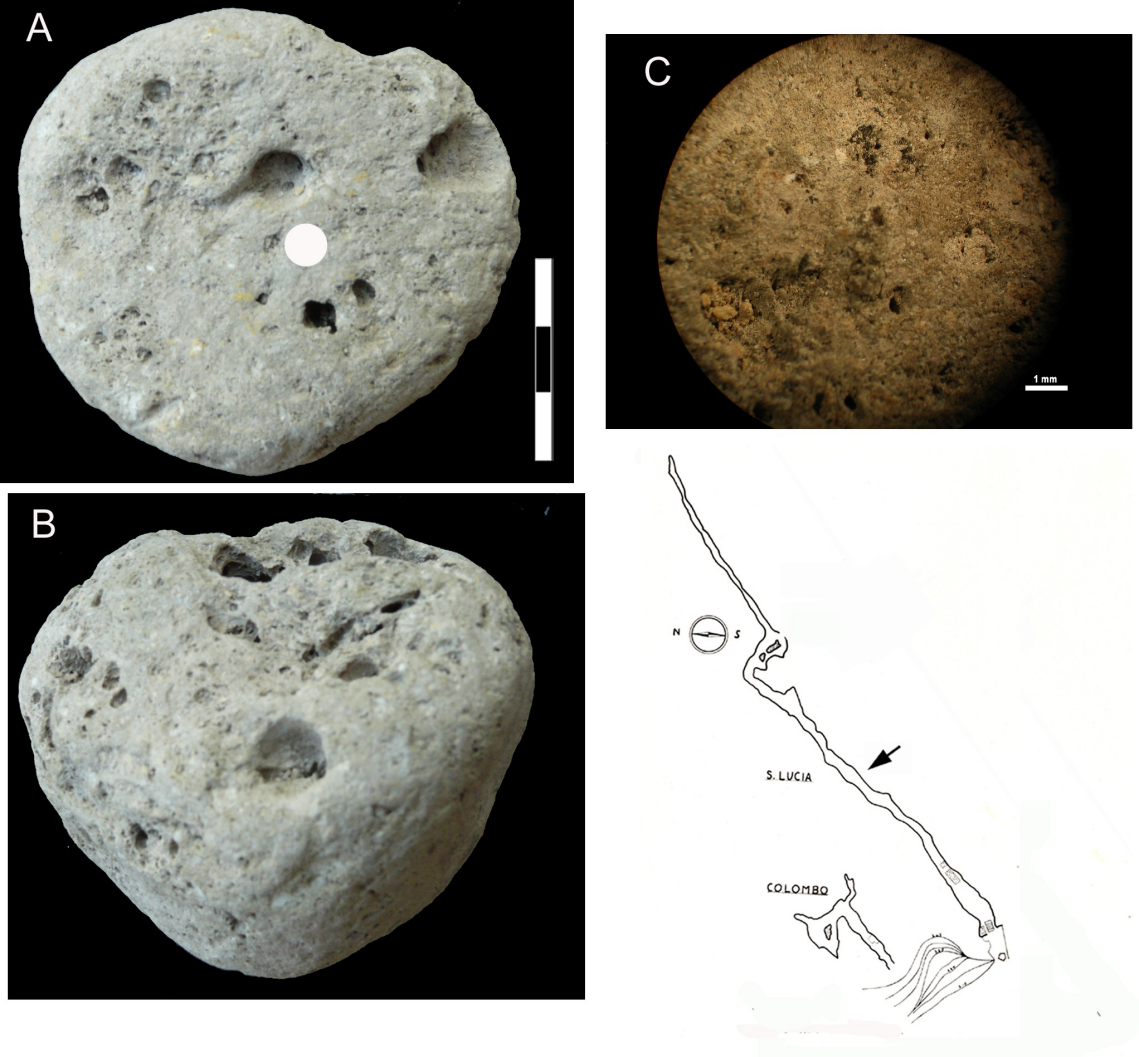
(A-B) Pumice from Santa Lucia cave (Toirano, Liguria, Italy). The white circle on A marks the area of the microscope photo in C showing the porous texture of the pumice. (D) Plan of the Santa Lucia cave, the arrow indicates the excavation area.

We have some evidence in favor of the second hypothesis based on a pumice from a Mousterian level of Grotta di Santa Lucia in Liguria (SLC thereafter) (Fig 13). The site is a narrow horizontal corridor about 280 m long. At the entrance there is a medieval shrine dedicated to Saint Lucia. The excavation by Carlo Tozzi [73] was done 46 m from the entrance (Fig 14). The cave is 4 km from the sea and 214 m above sea level. Therefore the pumice cannot have been transported by the sea or by wind but must have been collected by Neandertals on the beach.

The flat face of the Santa Lucia pumice might be the result of use as an abrader but there is only limited evidence of striations. Given the soft texture of the pumice, striations may have formed and be obliterated at the same time. There is no doubt that the Santa Lucia Neandertals collected pumices and they may have been aware of their abrasive qualities. In the Upper Paleolithic pumices were used as polishers, to polish points or needles [74]. Further support for collecting by Neandertals at Moscerini comes from two kinds of data:

- There are six pumices in the cave interior trench (Fig 2A) which is 15 m from the present dripline; wind going into a cave tends to lose energy.
- Pumices are not distributed randomly in the sequence (Fig 3). 71% of the pumices (35 of 49) are in layers 21–24 which are also the layers with the great majority of retouched shells (74.8%, that is 128 of 171) suggesting a connection between Neandertal gathering of natural objects from the beach.

The age of the Mousterian level at Santa Lucia is not known but it may be several millennia younger than Moscerini. Collection by Neanderthals is the most plausible hypothesis.

### Analysis of the Santa Lucia pumice

The pumice has a rounded shape and a smooth surface. It measures approximately 71x71x52 mm, it weighs 63 gm and has a volume of 106 cc. its specific gravity is 0.56 which falls in the common range of pumices [71]. The major and trace element composition of the pumice were determined using procedures described in the S1 File. The analytical results are reported in Table 10.

**Table 10:** Major- and trace-element composition of the pumice from Santa Lucia cave.

SiO_2_ (wt%)	60.73	Zr (μg/g)	479
TiO_2_	0.66	Nb	75
Al_2_O_3_	17.64	Mo	5.2
Fe_2_O_3_	2.85	Cs	18.2
MnO	0.14	Ba	16.3
MgO	0.57	La	84
CaO	1.21	Ce	184
Na_2_O	6.07	Pr	18.7
K_2_O	6.83	Nd	63
P_2_O_5_	0.20	Sm	11.1
LOI	3.10	Eu	1.33
Total	100.00	Gd	8.6
		Tb	1.36
Li (μg/g)	27.0	Dy	7.9
Be	12.3	Ho	1.59
V	31	Er	4.6
Cr	<1	Tm	0.71
Co	4	Yb	4.4
Ni	2	Lu	0.67
Cu	36	Hf	10.5
Zn	73	Ta	4.0
Ga	18.7	Tl	1.62
Rb	330	Pb	41
Sr	16.4	Th	37
Y	47	U	10.8

#### Results

**Fig 15:**
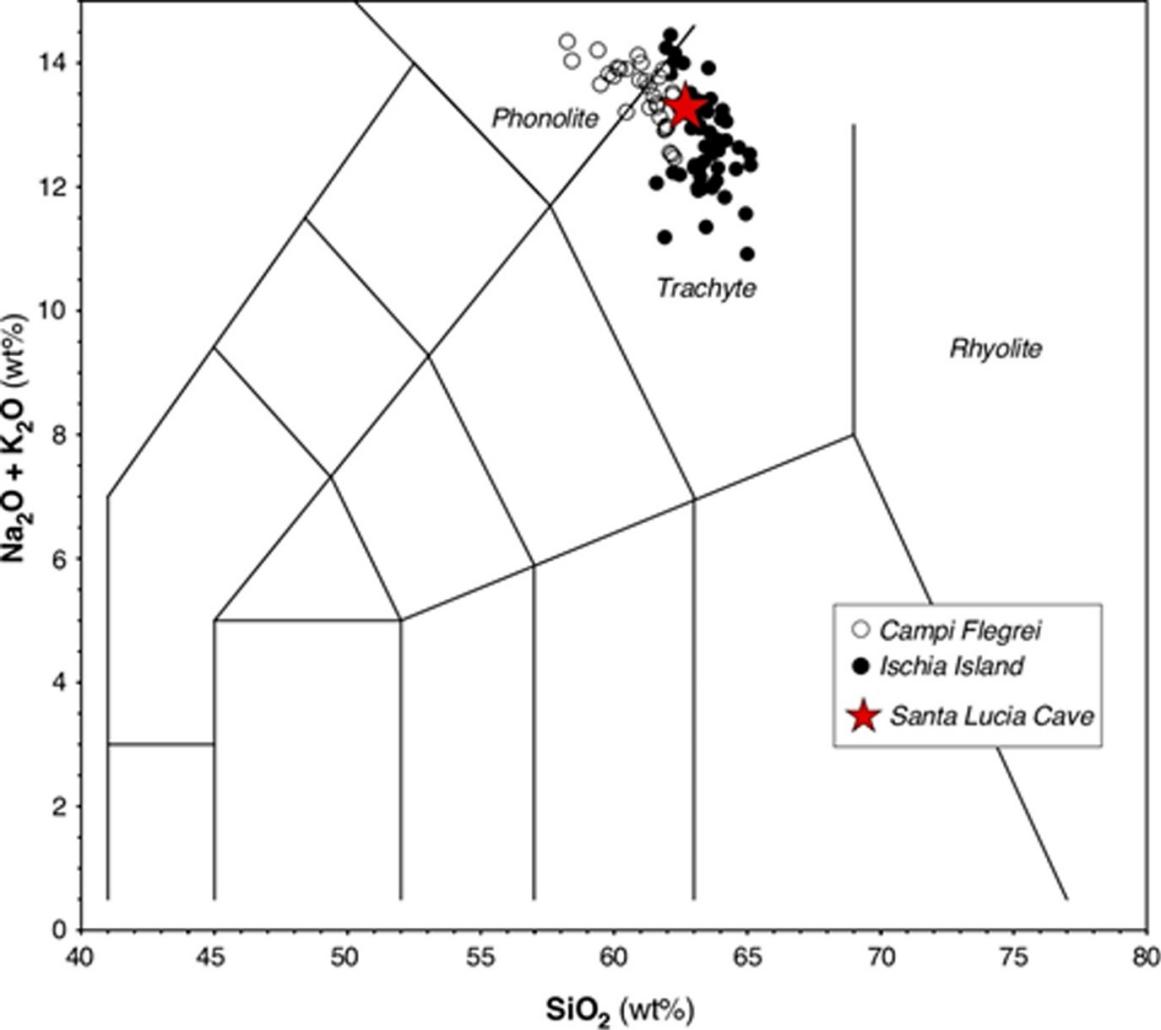
Total alkali vs silica diagram (TAS) for the Santa Lucia cave pumice and trachyte/phonolite rocks from the Campi Flegrei and the island of Ischia that have both Sr < 100 μg/g and Ba < 200 μg/g. For literature data on Campi Flegrei see [76–77]; on the island of Ischia see [78–79].

The SLC pumice has the composition of an alkali-rich trachyte. In the Total alkali vs silica classification diagram (Fig 15) [75] it falls in the trachyte field close to the phonolite field. It has a K_2_O/Na_2_O ratio of 1.1 and a moderately high LOI value (3 wt%). The most remarkable trace-element feature of this sample is the very low concentration of both Sr (16.4 μg/g) and Ba (16.3 μg/g) which indicates that this trachytic liquid suffered the extensive fractionation of feldspars. This is also in agreement with the marked negative Eu anomaly (Eu/Eu* = 0.41). Also peculiar are the high concentrations of the alkaline trace elements (Li, Rb, Cs) plus Zr, Pb, Th, U.

We made a systematic search of literature data on evolved products older than about 35 ka from central-western Mediterranean volcanoes. This lead to select a series of trachyte/phonolite pumices from the Campi Flegrei and the island of Ischia as the most similar to the SLC pumice. In particular, a small subset of samples from the Campi Flegrei and Ischia is populated by rocks with Sr < 40 μg/g and Ba < 50 μg/g (Fig 3A in S1 File). The volcanic rocks from Ischia belonging to this subset of samples (hereafter LowSrBa subset) are alkali trachyte and phonolite predating the eruption of the Green Tuff of Mount Epomeo, occurred about 56 ka ago [80]. Volcanic rocks from Campi Flegrei belonging to the LowSrBa subset are alkali trachyte and phonolite erupted during the Campanian Ignimbrite mega-eruption (dated to 39.85 ± 0.14 ka [70] and during the activity predating this eruption. In the Zr vs Rb plot (Fig 3B in S1 File) the SLC pumice plots inside the LowSrBa subset and close to samples both from Ischia and the Campi Flegrei.

In conclusion we can strongly support an origin of the SLC alkali trachyte pumice from the volcanoes of the Gulf of Naples. The most likely eruptions are those from Campi Flegrei or from the Island of Ischia which occurred before the Campanian Ignimbrite mega-eruption.

## Conclusions

Our research shows that only Italian assemblages and one occurrence in Greece show a systematic use of shells as tools. In fact, only two assemblages, from Moscerini and Cavallo caves, have a significant number of retouched shells, with 171 and 126 specimens each. At both sites only *Callista chione* shells were used and 23.9% of the specimens of Moscerini cave were collected as live animals from the sea floor. The archaeological evidence from Moscerini supports the idea that the abnormal bony growth of the external ear canal, quite common on Neandertal remains, indicates that Neandertals spent a significant amount of time collecting aquatic resources by skin diving [35–36]. Evidence of fishing in shallow waters is also documented by remains of freshwater fish at a Mousterian site in Italy (Castelcivita Cave; trout, chub, eel, NISP = 21) [38]. Additional evidence of freshwater fish consumption is provided by the sites of Payre in France [39] Kudaro in the Caucasus [41] Abri du Maras [40] dated to MIS 4; mostly cyprinids and percid, NISP = 167) and in Spain [43–44].

The presence of pumices in 17 archaeological layers is a surprising occurrence. The element composition shows that they derive from volcanic eruptions in the Ischia Island or from the Campi Flegrei. Their rounded edges indicate that they were transported by sea currents to the beach at the base of the Moscerini sequence. Their presence in the various layers of the cave sequence cannot be due to sea water that never reached the archaeological deposits. The most plausible hypothesis is that they were collected by Neandertals on the beach. Definitive evidence that Neandertals collected pumices is provided by a Mousterian layer in the Santa Lucia cave in Liguria.

This research shows that the exploitation of submerged aquatic resources and the collection of pumices, common in the Upper Paleolithic, were part of the Neandertal behavioral repertoire well before the arrival of modern humans in Western Europe. The technical competence, capacity for innovation and broad knowledge of the environmental resources have a greater time depth among non-modern humans than commonly acknowledged.

## Supporting information

S1 File Text, figures and tables. (PDF)

S2 File Lithic analysis. (PDF)

S3 File Permission to sample the pumice from Santa Lucia cave. (PDF)

S4 File Permission to publish two photos by B. Wilkens. (PDF)

S5 File Retouched shell tools. Exploratory experiment.
(PDF)
