# Supplementary material for: Neandertals on the beach: Use of marine resources at Grotta dei Moscerini (Latium, Italy)
Source: PLoS One. 2020 Jan 15;15(1):e0226690. doi: 10.1371/journal.pone.0226690 (PMC6961883; doi:10.1371/journal.pone.0226690)
Supplement: S1 File — (PDF) [file pone.0226690.s001.pdf]

# Supplementary Information

# Neandertals on the beach.
# Use of marine resources at Grotta dei Moscerini (Latium, Italy)

**Paola Villa*, Sylvain Soriano, Luca Pollarolo, Carlo Smriglio, Mario Gaeta, Massimo D'Orazio, Jacopo Conforti, Carlo Tozzi**

*** To whom correspondence should be addressed. E-mail: villap@colorado.edu**

## S1 File. Text, Figures and Tables

**Table 1. Taphnomy of retouched shell.** Four indeterminate cases (damaged or too broken) are excluded.

| State of preservation | N | % |
|---|---|---|
| Fresh | 166 | 99.4 |
| Slightly abraded | 1 | 0.6 |
| Abraded | 0 | 0 |
| Total | 167 | 100 |

**Other taphonomic features such as encrustation and dissolution can be pre- or post-burial (in the cave). We concentrated on features that would distinguish specimens gathered from the sea floor from beached pieces (Table 4 in the main text and associated discussion).**

**Table 2. Laterality of valves**

| Side of valve | N | % |
|---|---|---|
| Right valve | 38 | 22.2 |
| Left valve | 49 | 28.7 |
| Indetermined | 84 | 49.1 |
| Total | 171 | 100.0 |

The laterality of the valves can be determined considering a number of characters: the dorsal curvature, the orientation and width of growth lines, the orientation of the umbo or of portion of umbo, presence of hinge or parts of it, the position of the adductor muscles. We originally thought it may help us to determine a minimum number of individual shells but the high degree of fragmentation and the large number of indeterminate fragments defeated our purpose). If we use the total counts of *Callista* hinges by Stiner (1994, table 6.13, n= 182) the estimated MNI is 91.

# Analysis of the Moscerini cave pumices

## Analytical procedures

The pumices have been mounted in epoxy resin, ground flat at 30-40 µm of thickness and polished. The textural aspect and mineralogical assemblage of the pumice were analysed by optical microscope with the use of polarized light at the Earth Sciences Department, Sapienza University of Rome. Phase compositions were analysed at the CNR-Istituto di Geologia Ambientale e Geoingegneria di Roma, with a Cameca SX50 electron microprobe equipped with five wavelengths dispersive spectrometer (WDS). Quantitative analyses were performed using 15 kV accelerating voltage and 15 nA beam current. As standard we employed metals for Mn and Cr, Jadeite for Na, Wollastonite for Si and Ca, Orthoclase for K, Corundum for Al, Magnetite for Fe, Rutile for Ti and Periclase for Mg. Counting times were 20 s per element and 10 s for backgrounds. Light elements were counted first to prevent loss by volatilization. The PAP correction method was used. Minerals were analysed using a beam diameter of 1 µm whereas for glasses a defocused electron beam from 10 to 15 µm was used.

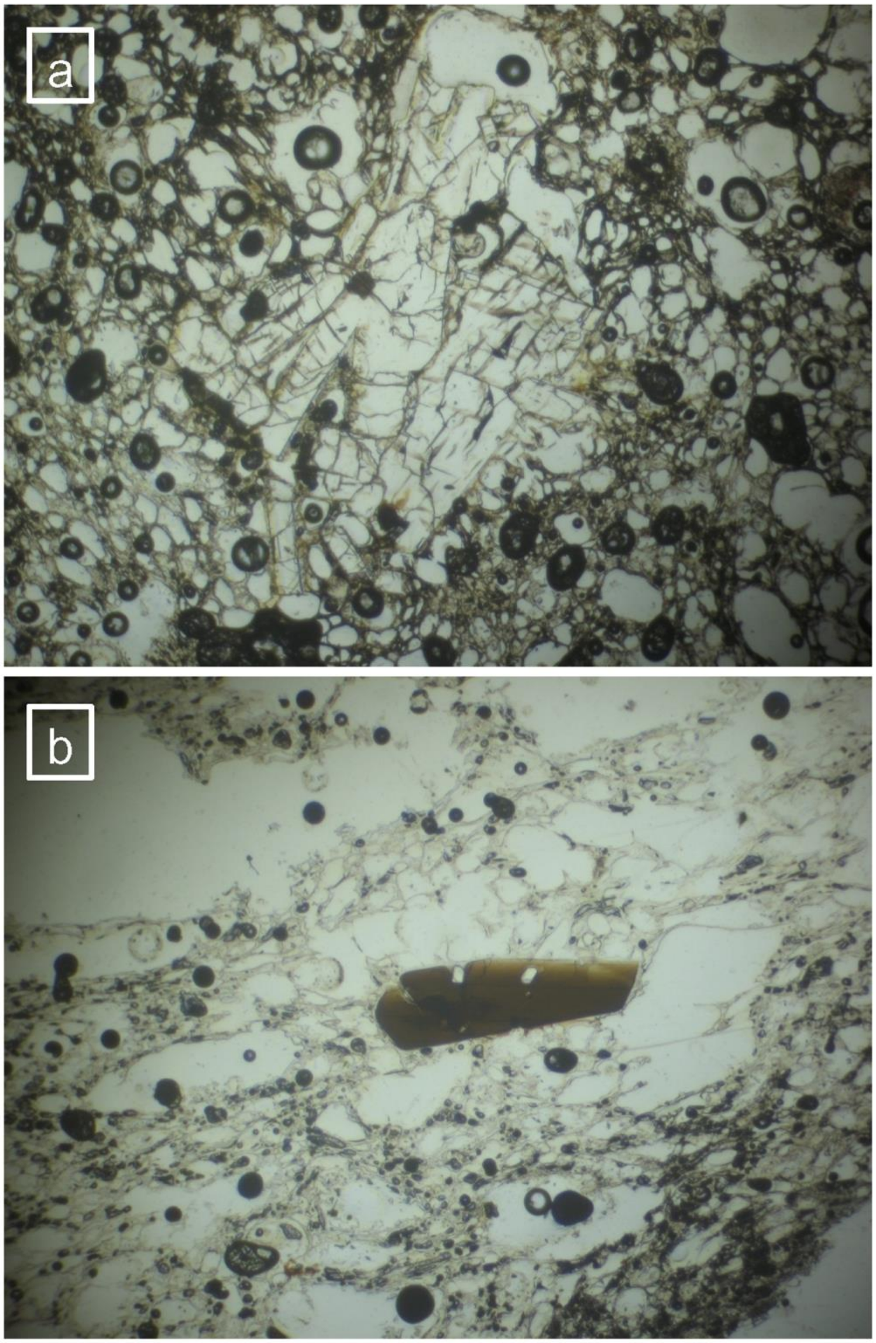


**Figure 2. Plane-polarized photomicrographs of the Moscerini cave pumices**: **(a)** cluster of feldspar phenocrysts (glomerocrysts) in the glassy vesiculated groundmass of pumice 37; (**b**) biotite phenocryst in the glassy vesiculated groundmass of pumice T3. The horizontal dimension of the photomicrographs is 2.5 mm.

## The pumice from Santa Lucia Cave. Analytical methods and results

The pumice is medium-grey colored, highly vesiculated and virtually free of phenocrysts. A small slice of the pumice was cleaned in ultrapure water and acetone and powdered by hand in

an agate mortar and pestle. Major elements were determined by X-ray fluorescence (XRF) on an ARL 9400 XP+ spectrometer using $LiBO_2/Li_2B_4O_7$ glass disks (sample:flux = 1:9). Estimated precision (relative standard deviation, RSD) is about 1% for $SiO_2$ and about 2% for the other major elements except those with low concentrations (~ < 0.50 wt%), for which the absolute standard deviation is about ± 0.01%. Loss on ignition (LOI) was determined by gravimetry at 1000 °C after preheating at 110 °C. The concentrations of 37 trace elements were determined by Inductively Coupled Plasma-Mass Spectrometry (ICP-MS) using a Perkin-Elmer NexION 300x spectrometer. About 50 mg of sample were dissolved with a mixture of concentrated HF + $HNO_3$ (Suprapur grade) on a hot plate. All analyses were performed at Pisa University's Dipartimento di Scienza della Terra. The analytical results are reported in Table 10.

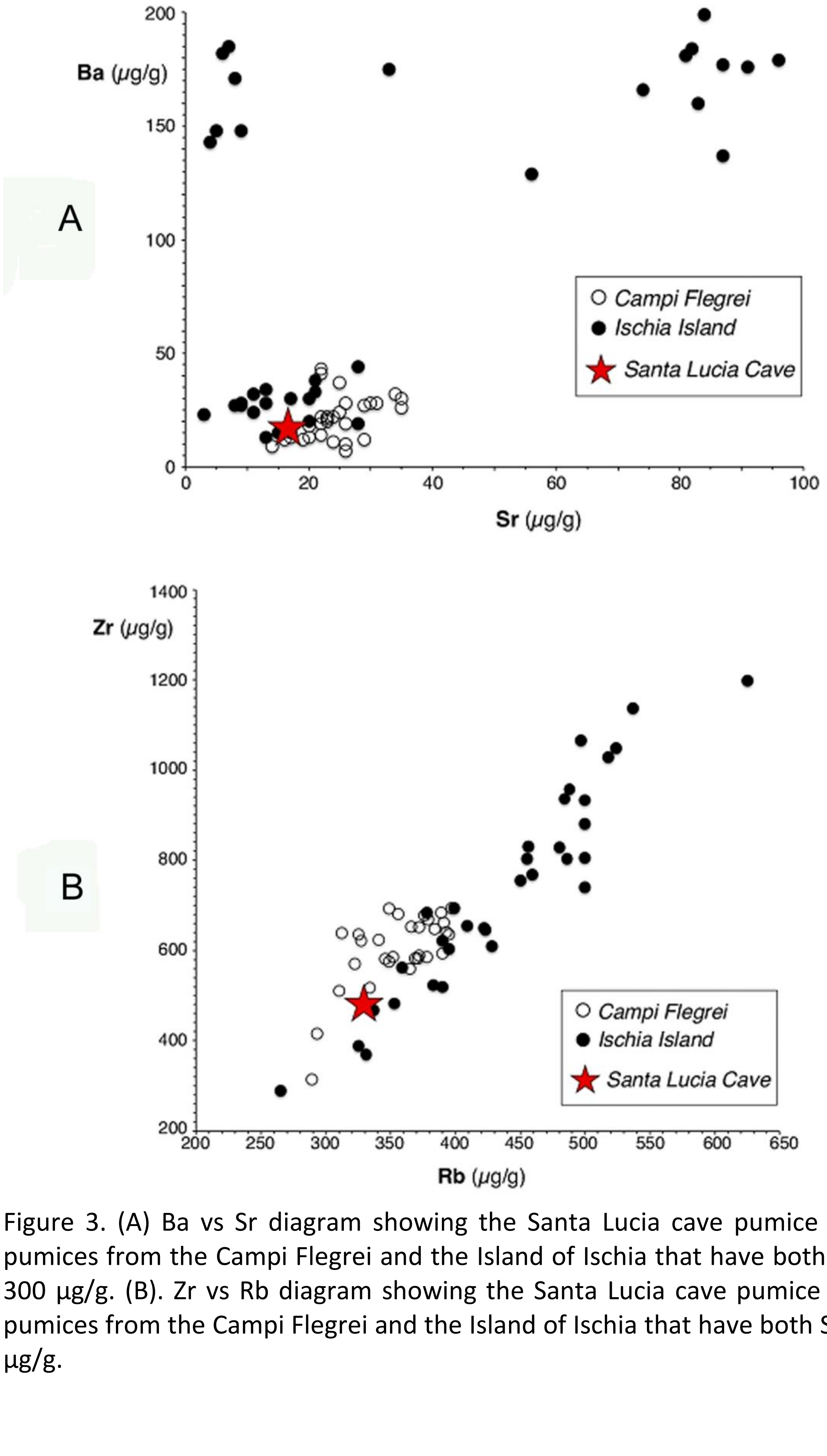


Figure 3. (A) Ba vs Sr diagram showing the Santa Lucia cave pumice and trachyte/phonolite pumices from the Campi Flegrei and the Island of Ischia that have both Sr < 100 μg/g and Ba < 300 μg/g. (B). Zr vs Rb diagram showing the Santa Lucia cave pumice and trachyte/phonolite pumices from the Campi Flegrei and the Island of Ischia that have both Sr < 40 μg/g and Ba < 50 μg/g.
