# Supplementary material for: Neandertals on the beach: Use of marine resources at Grotta dei Moscerini (Latium, Italy)
Source: PLoS One. 2020 Jan 15;15(1):e0226690. doi: 10.1371/journal.pone.0226690 (PMC6961883; doi:10.1371/journal.pone.0226690)
Supplement: S2 File — (PDF) [file pone.0226690.s002.pdf]

Supplementary Information

Neandertals on the beach.
Use of marine resources at Grotta dei Moscerini (Latium, Italy)

Paola Villa, Sylvain Soriano*, Luca Pollarolo, Carlo Smriglio, Mario Gaeta,
Massimo D'Orazio, Jacopo Conforti, Carlo Tozzi

*** To whom correspondence should be addressed. E-mail: sylvain.soriano@cnrs.fr;**

S2 File. Lithic analysis

This PDF file includes:

- Supplementary text: Description of lithic industry (production of blanks, retouched tools) in Layers 39-37, 26-20, 18-14 and INT1.
- Supplementary tables: Table 1 to Table 7
- Supplementary figures: Figure 1 to Figure 5

Description of lithic industry (production of blanks, retouched tools) in layers 39-37, 26-20, 18-14 and INT1

Our sample of lithic assemblages from Moscerini comprises 63 cores of which 28 are retouched, 403 flakes of which 183 are retouched, 7 retouched chunks, 18 tools retouched or shaped on pebble or pebble fragment, 27 blanks with double patina of which 26 are retouched, 13 retouched tools on undetermined blanks, 1 hammer stone, and 1 retoucher on a limestone pebble.

Debitage analysis

Almost 52% of the debitage is composed of ordinary flakes (cortical or not) that could not be associated with any system of production (S Table 2, S Fig. 3: C, F; S Fig. 4: I, L). They are not simply by-products since 45.5% of them are retouched. The frequency of retouched tools is almost the same within flakes with unidirectional parallel scars (48.9%) (S Fig. 2: H). Cores with negatives corresponding to such scar pattern represent 17.5% of the core total (S Table 3, S Fig. 3: J).

Production of blanks through 'Pontinian' bipolar flaking (*sensu* Soriano and Villa, 2017: fig. 16) is common (10.9% of flakes; 19.0% of cores). Fracture is non-conchoidal [wedging in (Cotterell and Kamminga, 1987)] so flakes and cores are usually undistinguishable then classified here as 'flakes', except when there are several negatives. Both flakes (S Fig. 3: G; S Fig. 4: J) and cores (S Fig. 2: E, K) with non-conchoidal fracture have been frequently retouched. Cores with a single invasive negative of removal but with a conchoidal fracture (S Fig. 3: A; S Fig. 4: K) could result from the same objective, namely to obtain the largest possible blank on these small flint pebbles. The frequency of Levallois debitage (0.7% of flakes; 6.3% of cores) is the lowest we have already observed in MP from Latium (Degano et al., 2019; Soriano and Villa, 2017; Villa et al., 2016). Nevertheless it is possible that some of the flakes we have classified as centripetal non-Levallois (2.7% of flakes) were actually produced on Levallois cores and that some Levallois blanks strongly modified by retouch cannot be identified.

Small tools

The toolkit (S Table 6) is dominated by scrapers (57.5%), mostly simple scrapers but double and transverse scrapers are also present, that is highly characteristic of the MP. Tools with converging retouched edges (points, convergent scrapers, including *déjeté* type) are well represented (15.6%) together with tools bearing irregularly retouched edges (12.7%) followed by the group of notches and denticulates. Vitagliano (1984) noted the general similarities between the Moscerini materials and Pontinian assemblages from other sites on the coast of Latium and this interpretation was not later discussed (Kuhn, 1995). The late MP industry from Fossellone layer 23 alpha was originally described as Pontinian (Blanc and Segre, 1953) and compared with Early MP industry from Torre in Pietra level d but now technologically characterized (Degano et al., 2019; Villa et al., 2016) emphasized strong differences with Moscerini. True bipolar debitage (i.e. on anvil) and discoidal debitage, so characteristic of Fossellone layer 23 alpha, are missing from Moscerini industries whereas Levallois debitage that is dominant at Torre in Pietra level d is very unusual at Moscerini.

Changes throughout the sequence

Ratios of the main technological features of the industries were calculated then represented with bar chart (S Fig. 5: B-H) to emphasize changes throughout the sequence. Phasing was done with respect to variations of amount of lithic pieces and shell tools in layers (S Fig. 5: A) and variations in cubic density of lithic (Text Fig. 5). In phase I (layers 39-37), there are several shell tools but lithic industry is more abundant. Phase II (from layer 26 to 22) is characterized by shells tools outnumbering lithic pieces. Within phase III, the amount of lithic pieces rises whereas shell tools are randomly present. In phase IV (layer INT1 but unstudied layers 13 to 1 and INT4 to INT are also concerned), shell tools practically disappear.

By many aspects layers from phase II differ from others layers from our sample. Shell tools are much more frequent than retouched stone tools (S Fig. 5: B). It is important to note that the greater amount of retouched stone is observed in this phase II (S Fig. 5: C). Phases I to III have in common a high debitage to core ratio (S Fig. 5: D) suggesting that at least a part of flakes were produced outside from the site and introduced as unretouched blanks or retouched tools rather than a highest productivity because raw materials are small sized and cores are usually bearing three or less than three negatives greater than 10 mm. In phase IV (layer INT1), the debitage to core ratio is more in agreement with production of flakes within the site. It is in phase II that non-local grey to black flint (S Fig. 1: 1, 2, 5) are more frequently used (S Fig. 5: E). The provenience of these exotic raw materials is still unknown but S. Kuhn (1995, p. 45) suggested that primary outcrops of flint can be found 50-60 km inland thus nearer than the Monte Genzana flint source. This high frequency of non-local flint is unusual in MP occupations from the Latium where raw materials were almost exclusively collected in the beach deposits (Ansuini et al., 1991; Caputo et al., 2001; Kuhn, 1995; Riel-Salvatore and Negrino, 2009) or at least in fluvial gravels (Soriano and Villa, 2017). The proportion of retouched stone tools or blanks with double patina in phase II is the highest (S Fig. 5: F) indicating an opportunistic reuse of older stone tools. The closest source of patinated tools is the site itself perhaps in older layers exposed by erosion on the sloping deposits front of the cave, now dispersed. Collecting older patinated stone tools cannot be considered an unambiguous response to raw material supply shortage because it is not in phase II but in the following phase III that the highest occurrence of tool recycling and reworking is noticed (S Fig. 5: G).

'Pontinian' bipolar flaking as described by (Villa et al., 2016) was identified in some industries from Latium (Torre in Pietra level d, Sant'Agostino, Guattari lower levels) but is lacking or is infrequent in others (Fossellone layer 23 alpha, Grotta Barbara, Guattari upper levels) (Degano et al., 2019; Soriano and Villa, 2017; Villa et al., 2016 and pers. obser.). At Moscerini the lowest use of this kind of production is recorded in phase II (S Fig. 5:H).

<u>Non-local raw material.</u> The flint types we hypothesized to be non-local at Moscerini have never been observed in any of the other LP/MP industry of the Latium we have studied to date. Non-beached cortex are dominant on this flint types thus excluding a local source because marine fossil beaches are the only source of flint in the coastal plain and around the Monte Circeo according to Kuhn [3 : p.44]. A non-local provenience is supported by the techno-economical pattern of the part of the assemblage made from these types of flint (low dorsal cortex, low frequency of cores, high frequency of flakes from tool reworking and tool recycling, high

frequency of Kombewa flakes compared to local flint), as expected in MP context for lithics coming from afar, used for a long time and introduced in the site as retouched tools or recycling products (Geneste J-M. 1992).

Supplementary tables

Table 1. Grotta dei Moscerini. Distribution of cortex type relative to raw material type for all layers of our sample (Layers 39-37, 26-20, 18-14 and INT1; undetermined cortex type excluded).The non-local flint category includes grey-blue chalcedony-like flint, light brown to dark brown semi-translucent flint and a grey to black, translucent or semi-translucent flint. The local flint category includes various types of opaque flint, from pale grey..

	With cortex					Without cortex	
	Fresh cortex	Natural surface	Abraded	Rolled	Total with cortex		Total
Non-local flint	4	19	1	2	26	69	95
	4.2%	20.0%	3.2%			72.6%	100%
Local flint	13	21	11	265	310	99	409
	3.2%	5.1%	67.5%			24.2%	100%
	4.2%	6.8%	89.0%		100%		
Jasper	0	0	0	1	1	1	2
Quartzite	0	1	0	3	4	1	5
Chert	0	0	0	5	5	1	6
Limestone	1	0	0	11	12	0	12
Undetermined	0	0	0	2	2	1	3
Total					360		532

Note. Rolled = very abraded

Table 2 A. Grotta dei Moscerini. Counts of flake types by layer

	Layers															
Flake type	INT1	14	15	16	17	18	20	21	22	23	24	25	26	37	38	39
Cortical flake (>75% cortex)	6	2	5	5	4	6	5	6		1			3	4	1	3
Partly cortical ordinary flake[a]	12	7	4	6	14	7	6	9	1	3	2		2	6	1	3
Non cortical ordinary flake	4	2	6	7	9	11	12	11	1	3	2	4		2	1	2
Flake with unidirectional parallel scars	6	3	3	2	5	3	5	6	2	1	2		1	2	1	5
Levallois flake	1	1			1											
Centripetal (non-Levallois) flakes	2		1	1	2	2	1	1	1							
Pseudo-Levallois flakes	1			1	1			1						2		
Kombewa flake (1st or 2nd generation)			1	5	3		2	3		2	1			3		1
Flake with non conchoidal fracture >50% cortex	5	2	1	4	5	4	5	2						2	6	2
Flake with non conchoidal fracture <50% cortex	2			2					1				1			
Flake from tool making	1	1	1	3	4	4	2		1	1				1		1
Flake from tool reworking and tool recycling		1		1	4	1		1	2				1	1		
Accidental flake from hammerstone								1								
Other types of flakes					1			1	1		1					1
Undetermined flake	1	1	2	1	2	2	3	1	4	2		1				2
Total	41	20	24	38	55	40	41	43	14	13	8	5	8	23	10	20

Table 2B. Grotta dei Moscerini. Counts of retouched and unretouched flakes (Layers 39-37, 26-20, 18-14 and INT1). Flakes with double patina are not included.

**Flake type**	**Retouched**	**Unretouched**	**Total**	**%**
Cortical flake (>75% cortex)	26	25	51	*12.7*
Partly cortical ordinary flake[a]	45	38	83	*20.6*
Non cortical ordinary flake	25	52	77	*19.1*
Flake with unidirectional parallel scars	23	24	47	*11.7*
Levallois flake	1	2	3	*0.7*
Centripetal (non-Levallois) flakes	4	7	11	*2.7*
Pseudo-Levallois flakes	2	4	6	*1.5*
Kombewa flake (1st or 2nd generation)	6	15	21	*5.2*
Flake with non conchoidal fracture >50% cortex	20	18	38	*9.4*
Flake with non conchoidal fracture <50% cortex	4	2	6	*1.5*
Flake from tool making	1	19	20	*5.0*
Flake from tool reworking and tool recycling	3	9	12	*3.0*
Accidental flake from hammerstone	0	1	1	*0.2*
Other types of flakes	2	3	5	*1.2*
Undetermined flake	21	1	22	*5.5*
Total	183	220	403	*100*
	45.4%	54.6%	100%	

[a] "Ordinary" flakes are the generic product of any kind of core.

Table 3A. Grotta dei Moscerini. Counts of cores by layer

	Layers																
Core type	INT1	14	15	16	17	18	20	21	20+21	22	23	24	25	26	37	38	39
Levallois core / possible Levallois core				2	1			1									
Core with series of unidirectional parallel removals	2	2		1	1	1	1	1	1	1							
Core with centripetal removals (non-Levallois)	1	1		1	1												
Core on flake	2	2	2	1										1			
Core with a single invasive removal	2			1	3		1	1									
Core with removals bearing non conchoidal fracture	3	1	2	2				2							1		1
Tested raw material		1								1			1				1
Other type of core	2				1			1		1	1						1
Core of undetermined type	2							1								2	
Total	14	7	4	8	7	1	2	7	1	3	1	0	1	1	1	2	3

Table 3B. Grotta dei Moscerini. Counts of retouched and unretouched cores (Layers 39-37, 26-20, 18-14 and INT1).

**Core type**	**Retouched**	**Unretouched**	**Total**	**%**
Levallois core or possible Levallois core	0	4	4	*6,3*
Core with series of unidirectional parallel removals	6	5	11	*17,5*
Core with centripetal removals (non-Levallois)	3	1	4	*6,3*
Core on flake	1	7	8	*12,7*
Core with a single invasive removal	3	5	8	*12,7*
Core with removals bearing non conchoidal fracture	10	2	12	*19,0*
Tested raw material	0	4	4	*6,3*
Other type of core	1	6	7	*11,1*
Core of undetermined type	4	1	5	*7,9*
Total	28	35	63	*100*
	44.4%	55.6%	100%	

Table 4. Frequency of retouched blanks in Middle Paleolithic industries from Latium. The sorting and analysis procedures were the same for these industries (Villa et al., 2016; Degano et al., 2019).

	Unretouched	Retouched
Moscerini (Layers 39-37, 26-20, 18-14 and INT1)	48.2%	51.8%
Torre in Pietra, level d	62.1%	37.9%
Fossellone level 23 alpha	64.9%	35.1%
Sant'Agostino layer A1	76.2%	23.8%

Table 5. Grotta dei Moscerini. Blanks of retouched tools (Layers 39-37, 26-20, 18-14 and INT1).

**Categories**	**N**	**%**
Flakes and flake fragments	183	66.5
Cores and core fragments	28	10.2
Chunks	7	2.5
Pebble or pebble frag.	18	6.5
With double patina	26	9.5
Undetermined	13	4.7
Total	275	100

Table 6. Grotta dei Moscerini. Composition of retouched tools (Layers 39-37, 26-20, 18-14 and INT1).

**Tool types**	**N**	**%**
Scrapers	158	57.5
Points and convergent scrapers	43	15.6
Notches, denticulates and beaks	19	6.9
Bifacially retouched tools	6	2.2
Partial, discontinuous or irregular retouch	35	12.7
Tool fragment	11	4.0
Utilized	3	1.1
	275	100

Table 7. Grotta dei Moscerini. Data on lithic density used in Fig. 5. Layer thickness are from unpublished stratigraphic descriptions from A. Segre except for those in red which were measured on the unpublished section from A. Segre. n.a.: non available data.

Layer	Count of lithic IIPU Anagni	Pigorini museum	Total lithic	Layer thickness (m)	Excavated surface (m²)	Density of lithic per m^3
1	3	0	3	0,30	3,00	3,33
2	15	5	20	0,30	3,00	22,22
3	13	2	15	0,30	3,00	16,67
4	0	0	0	0,30	3,00	0,00
5	2	0	2	0,35	3,00	1,90
6	1	0	1	0,25	3,00	1,33
7	0	0	0	0,20	3,00	0,00
8	1	0	1	0,30	3,00	1,11
9	2	0	2	0,18	3,00	3,70
10	2	0	2	0,20	3,00	3,33
11	3	1	4	0,20	3,00	6,67
12	1	1	2	0,15	3,00	4,44
13	12	2	14	0,30	3,00	15,56
14	43	3	46	0,15	3,00	102,22
15	36	4	40	0,20	3,00	66,67
16	58	4	62	0,25	3,00	82,67
17	90	6	96	0,25	3,00	128,00
18	71	3	74	0,15	3,00	164,44
19	26	5	31	0,10	3,00	103,33
20	69	4	73	0,15	3,00	162,22
21	80	4	84	0,15	3,00	186,67
22	28	1	29	0,20	3,00	48,33
23	16	1	17	0,20	3,00	28,33
24	9	1	10	0,20	3,00	16,67
25	7	2	9	0,15	3,00	20,00
26	11	3	14	0,20	3,00	23,33
27	12	0	12	0,15	3,00	26,67
28	12	1	13	0,20	3,00	21,67
29	1	1	2	0,15	3,00	4,44
30	14	1	15	0,20	3,00	25,00
31	18	4	22	0,20	3,00	36,67
32	27	3	30	0,20	3,00	50,00
33	31	3	34	0,20	3,00	56,67
34	21	1	22	0,15	3,00	48,89
35	10	3	13	0,24	3,00	18,06
36	8	1	9	0,24	3,00	12,50
37	31	4	35	0,05	3,00	233,33
38	15	2	17	0,40	3,00	14,17
39	50	0	50	0,60	3,00	27,78
40	0	0	0	0,35	3,00	0,00
41	7	0	7	0,60	3,00	3,89
Subtotal	856	76	932			

1-5	4	0	4		
12-17	36	0	36		
int.	12	0	12	n.a.	n.a.
int. 1	76	1	77	n.a.	n.a.
int. 1a	6	0	6	n.a.	n.a.
int. 1b	23	0	23	n.a.	n.a.
int. 1c	3	0	3	n.a.	n.a.
int. 1d	33	0	33	n.a.	n.a.
int. 2	27	0	27	n.a.	n.a.
int. 3	71	0	71	n.a.	n.a.
int. 4	32	8	40	n.a.	n.a.
Subtotal	283	9	292		
Total	1139	85	1224		

Supplementary figures

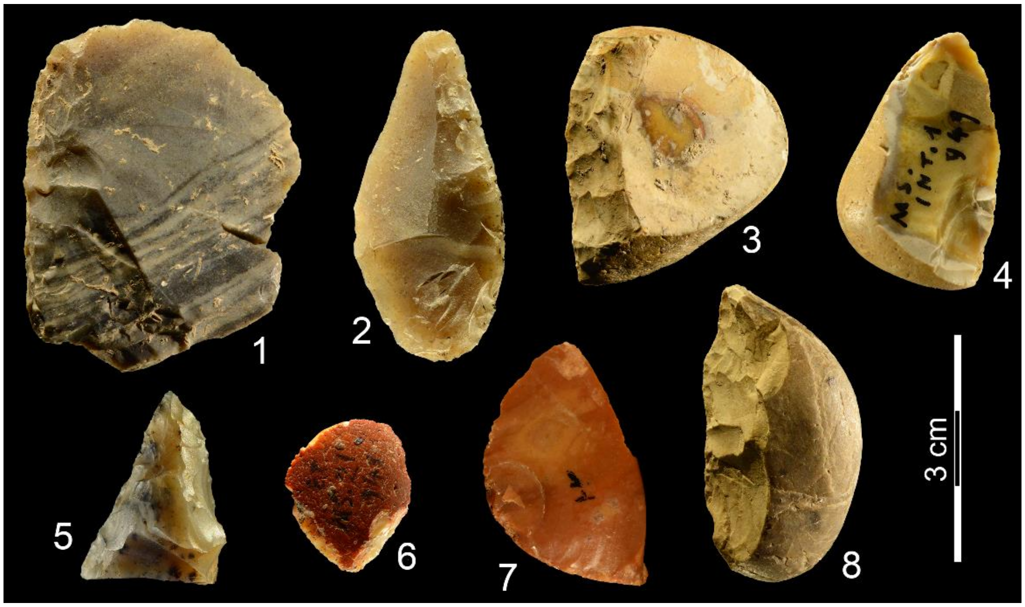

Figure 1. Grotta dei Moscerini. Examples of local (3, 4, 6-8) and non-local (1, 2, 5) types of flint. Photos P. Villa.

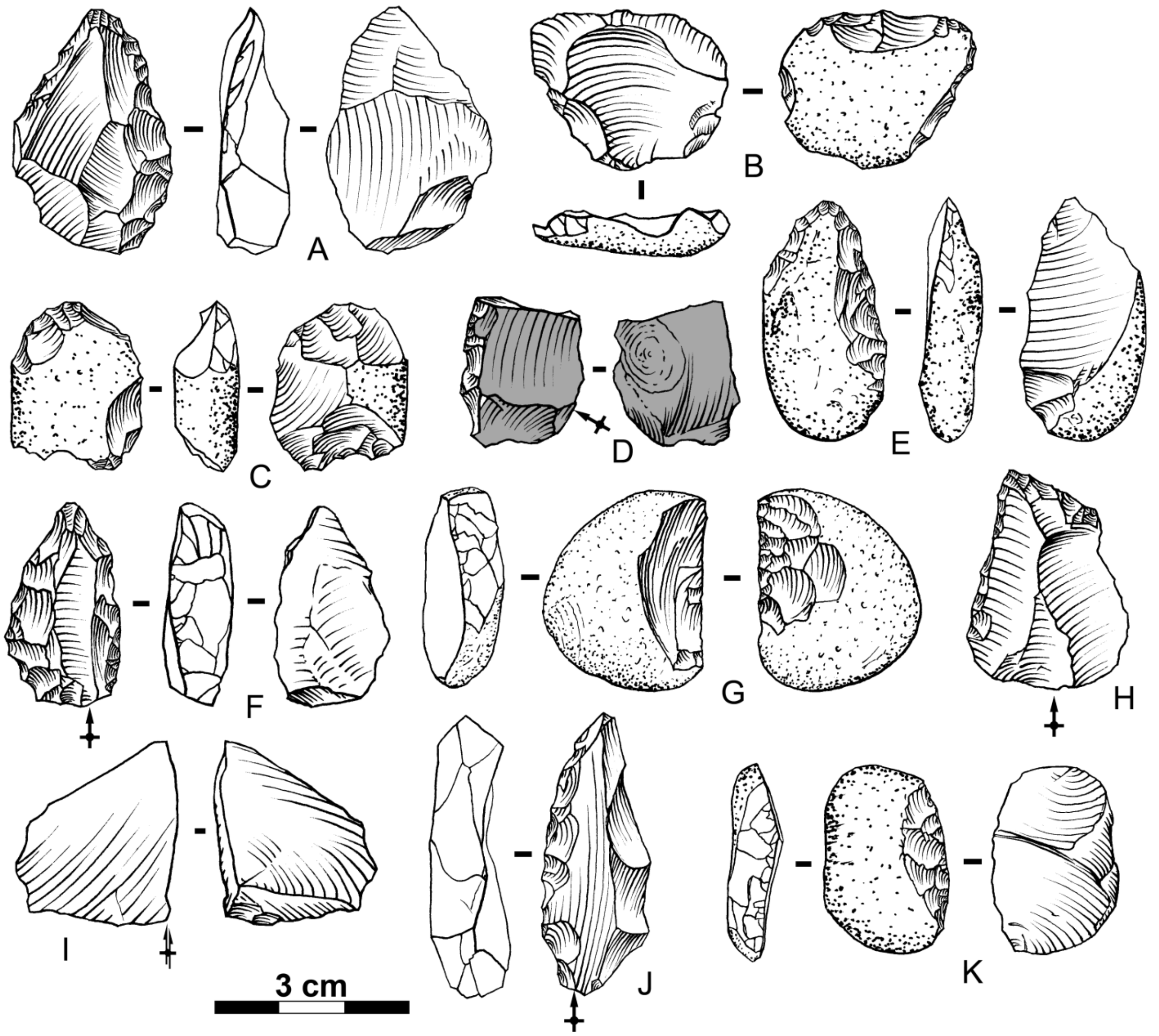

Figure 2. Grotta dei Moscerini. Stone artifacts from layers 20 (A, C), 21 (B, E), 22 (D, F, H, I), and 37 (G, J, K). (A) Double scraper (on a recycled core?); (B) Levallois core?; (C) Bifacial scraper shaped on a pebble; (D) Sidescraper on a recycled patinated flake (Surfaces shaded in grey); (E) Convergent scraper on a core with a single invasive removal; (F) Convergent scraper on a flake; (G) Bifacial scraper shaped on a pebble; (H) Convergent scraper on a flake with scars of unidirectional removals; (I) Kombewa flake with Siret fracture; (J) Sidescraper on a flake coming from a core on flake (only a small part of the ventral surface is remaining on the dorsal surface of the secondary flake); (K) Sidescraper on a 'Pontinian' bipolar core (*sensu* Soriano and Villa, 2017, fig. 16). Drawings S. Soriano.

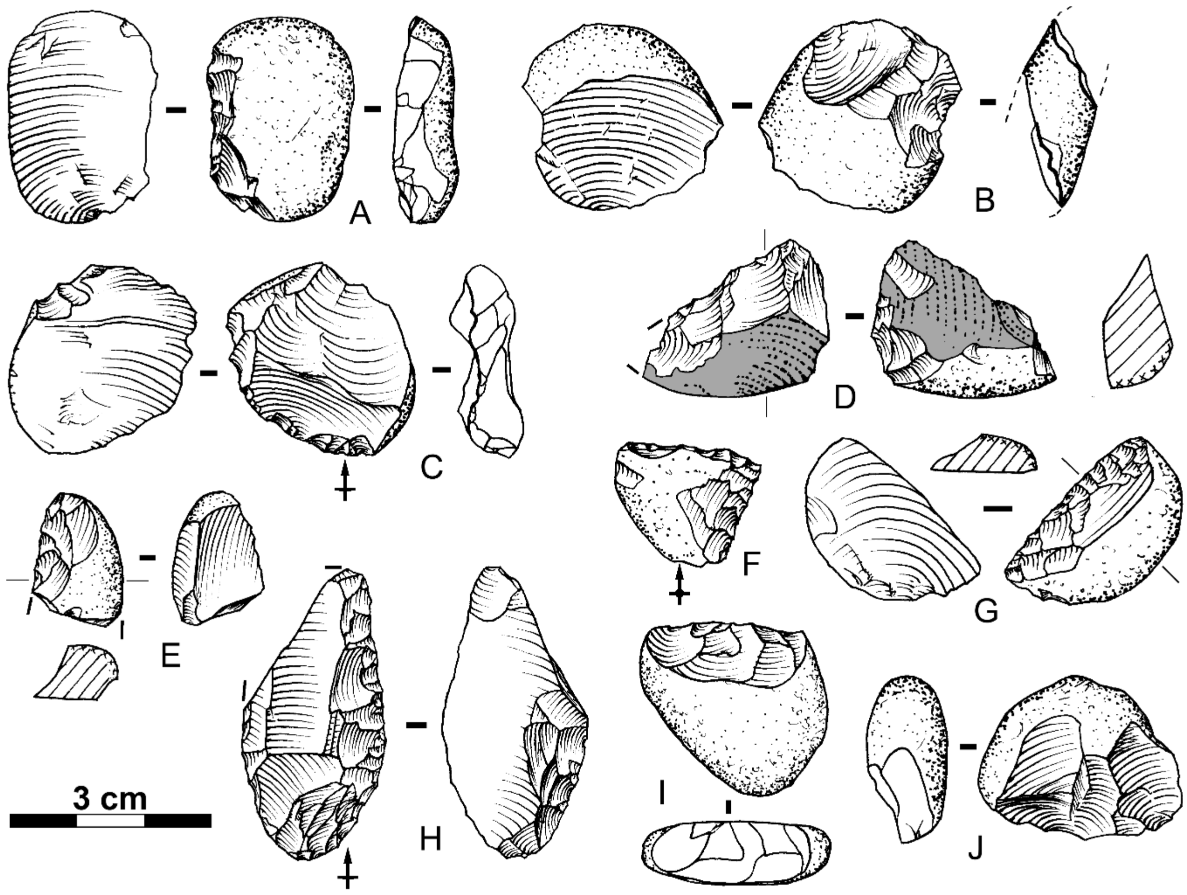

Figure 3. Grotta dei Moscerini. Stone artifacts from layer INT1 except (B), from layer INT. (A) Sidescraper on a core with a single invasive removal; (B) Core; (C) Sidescraper on a flake; (D) Sidescraper on a recycled patinated flake (Surfaces shaded in grey); (E) Sidescraper on recycled a core; (F) Déjeté scraper (micro-point) on a cortical flake; (G) Sidescraper on a 'flat' semi-cortical flake (*sensu* Soriano and Villa, 2017, fig. 16); (H) Sidescraper with thinned back on (Levallois?) flake; (I) Unifacial scraper shaped on a pebble; (J) Unipolar core. Drawings S. Soriano.

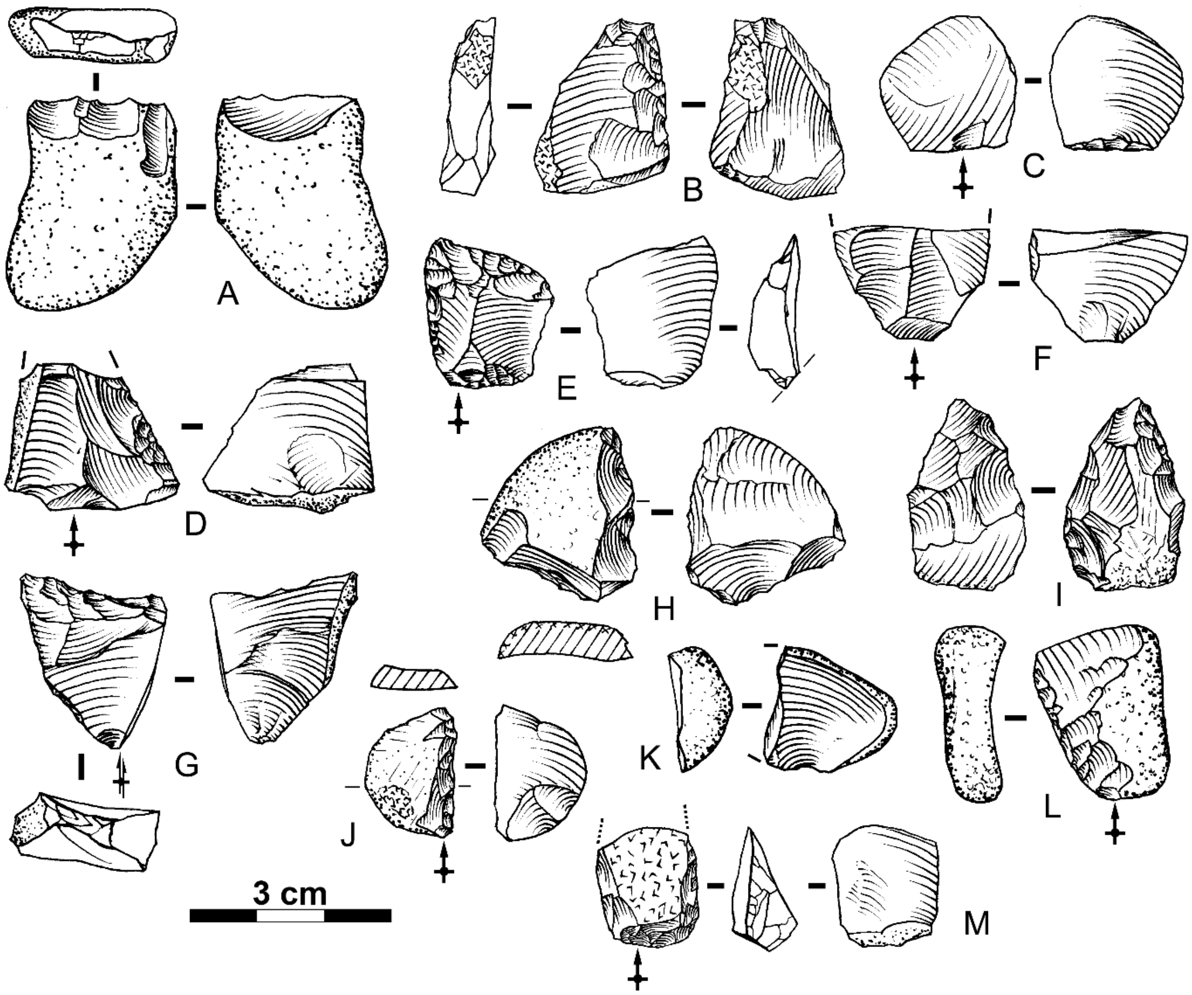

S

Figure 4. Grotta dei Moscerini. Stone artifacts from layer 17 except (A), from layer 15 and (M) from layer 18. (A) Bifacial scraper shaped on a pebble; (B) Bifacial scraper (on a recycled core?), burnt; (C) Kombewa flake; (D) Sidescraper on a flake; (E) Déjeté scraper (micro-point) on a retouch flake; (F) Flake coming from a core on flake (only a small part of the ventral surface is remaining on the dorsal surface of the secondary flake); (G) Overshot flake removed transversely on the dorsal surface of a double sidecraper with alternate retouch; (H) Denticulate sidescraper on a recycled core; (I) Partly bifacial point shaped on a flake; (J) Sidescraper on a 'flat' cortical flake (*sensu* Soriano and Villa, 2017, fig. 16) with basal thinning; (K) core with a single invasive removal; (L) Sidescraper on semi-cortical flake; (M) Broken (burnt) double sidescraper on a flake. Drawings S. Soriano.

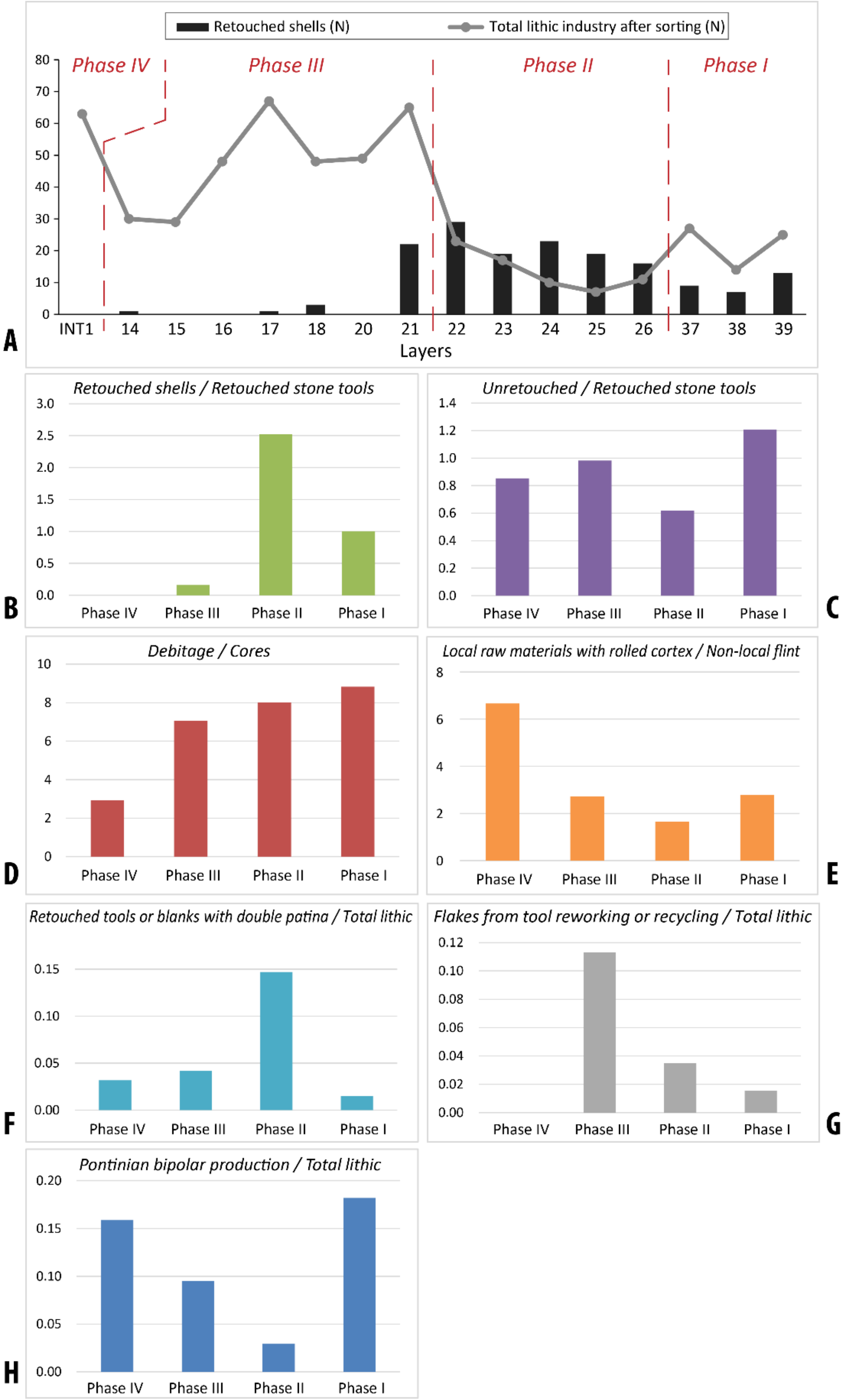

Figure 5. Grotta dei Moscerini. (A) Phasing of the archaeological sequence with respect to variations of amount of lithic pieces and shell tools in layers of our sample (Layers 39-37, 26-20, 18-14 and INT1); (B-H) Ratios of the main technological features of the industries.

References

Ansuini, P., La Rosa, M., Zei, M., 1991. Open Air Mousterian Sites In Central-South Coastal Latium. Quaternaria Nova 1, 479–498.

Blanc, A.C., Segre, A.G., 1953. La Grotta del Fossellone (Grotte du Grand Fossé), in: Blanc, A.C., Segre, A.G. (Eds.), Excursion Au Mont Circé. Le Volcan Latial – Le Mont Circé. INQUA, IVe Congrès International, Rome, Pise, 1953. INQUA, Rome, pp. 37–85.

Caputo, C., Arnoldus-Huyzendveld, A., Pugliese, F., 2001. The Roman area natural environment: geomorphological features and lithic resources, in: Cavarretta, G., Gioia, P., Mussi, M., Palombo, M.R. (Eds.), La terra degli elefanti: atti del 1. Congresso internazionale ; Roma, 16-20 ottobre 2001 ; proceedings of the 1st International congress. Consiglio nazionale delle ricerche, Roma.

Cotterell, B., Kamminga, J., 1987. The Formation of Flakes. Am Antiq 52, 675–708. https://doi.org/10.2307/281378

Degano, I., Soriano, S., Villa, P., Pollarolo, L., Lucejko, J.J., Jacobs, Z., Douka, K., Vitagliano, S., Tozzi, C., 2019. Hafting of Middle Paleolithic tools in Latium (central Italy): New data from Fossellone and Sant'Agostino caves. PLOS ONE 14, e0213473. https://doi.org/10.1371/journal.pone.0213473

Geneste, J.M., 1992. L'approvisionnement en matières premières dans les systèmes de production lithique : la dimension spatiale de la technologie. *Treballs d'arqueologia*, (1), 1-36.

Kuhn, S.L., 1995. Mousterian Lithic Technology. Princeton University Press, Princeton.

Riel-Salvatore, J., Negrino, F., 2009. Early Upper Paleolithic population dynamics and raw material procurement patterns in Italy, in: Camps, M., Szmidt, C. (Eds.), The Mediterranean from 50,000 to 25,000 BP: Turning Points and New Directions. Oxbow Books, Oxford, pp. 211–230.

Soriano, S., Villa, P., 2017. Early Levallois and the beginning of the Middle Paleolithic in central Italy. PLoS ONE 12, e0186082. https://doi.org/10.1371/journal.pone.0186082

Villa, P., Soriano, S., Grün, R., Marra, F., Nomade, S., Pereira, A., Boschian, G., Pollarolo, L., Fang, F., Bahain, J.-J., 2016. The Acheulian and Early Middle Paleolithic in Latium (Italy): Stability and Innovation. PLoS One 11, e0160516. https://doi.org/10.1371/journal.pone.0160516

Vitagliano, S., 1984. Nota sul Pontiniano della Grotta dei Moscerini, Gaeta (Latina), in: XXIV Riunione Scientifica Dell'Istituto Italiano Di Preistoria e Protostoria Nel Lazio, 8-11 Oct. 1982. Istituto Italiano di Preistoria et Protostoria, Firenze, pp. 155–164.
