# Supplementary material for: Neandertals on the beach: Use of marine resources at Grotta dei Moscerini (Latium, Italy)
Source: PLoS One. 2020 Jan 15;15(1):e0226690. doi: 10.1371/journal.pone.0226690 (PMC6961883; doi:10.1371/journal.pone.0226690)
Supplement: S3 File — (PDF) [file pone.0226690.s003.pdf]

Supplementary Information

Neandertals on the beach.
Use of marine resources at Grotta dei Moscerini (Latium, Italy)

Paola Villa*, Sylvain Soriano, Luca Pollarolo, Carlo Smriglio, Mario Gaeta, Massimo D'Orazio, Jacopo Conforti, Carlo Tozzi

*** To whom correspondence should be addressed. E-mail: villap@colorado.edu**

S3 File. Permission to sample the Santa Lucia pumice.

Genova, 10. 04. 2019

A Prof. Carlo Tozzi
Università di Pisa
tozzi@arch.unipi.it

Ministero per i Beni e le Attività Culturali

DIREZIONE GENERALE ARCHEOLOGIA BELLE ARTI E PAESAGGIO

SOPRINTENDENZA ARCHEOLOGIA, BELLE ARTI E PAESAGGIO PER LA CITTÀ METROPOLITANA DI GENOVA E LE PROVINCE DI IMPERIA, LA SPEZIA E SAVONA

Prot. MBAC-SABAP-LIG ..8383..

Cl. 34.04.07/1.2

Allegati

OGGETTO: TOIRANO, GROTTA DI SANTA LUCIA SUPERIORE
ART21c1b, ART21c4, ART107c1, ART108c3-3bis
D. Lgs. 42/2004 Codice dei Beni culturali e del Paesaggio, art.21, c1b: *autorizzazione allo spostamento, anche temporaneo, di beni culturali mobili*; c.4: *autorizzazione ad opere e lavori*; art.107 c1: *uso strumentale e precario e riproduzione di beni culturali*; art.108 c3-3bis: *canoni di concessione, corrispettivi di riproduzione, cauzione.*
Richiesta autorizzazione di studio ciottolo in pomice proveniente da livelli musteriani - scavi 1962.
Richiedente: Prof. Carlo Tozzi, Università di Pisa

A RISCONTRO della richiesta inoltrata dalla S.V. il 01/04/2019, assunta al protocollo col n. 7520 in data 02/04/2019, volta ad ottenere l'autorizzazione in oggetto e delle comunicazioni inoltrate per le vie brevi;

AI SENSI di quanto previsto dall'art. 21 D. Lgs 22.01.2004 n° 42 e ss.mm.ii., *Codice dei Beni Culturali e del Paesaggio;*

VALUTATE le finalità e l'importanza delle indagini richieste;

VALUTATO altresì che le analisi previste, allo stato attuale delle conoscenze, risultano essere compatibili con le esigenze di tutela del bene culturale in oggetto;

QUESTA SOPRINTENDENZA AUTORIZZA

1. Per quanto di competenza, l'accesso del richiedente e della Prof.ssa Paola Villa della University of Colorado al reperto in oggetto in deposito presso il Comune di Toirano. Tempi e modalità andranno concordati direttamente con l'ente interessato, con l'ausilio della dott.ssa Marta Zunino, responsabile scientifico in servizio presso le Grotte di Toirano.
2. Il trasporto presso l'Università di Pisa e il prelievo di un campione per analisi petrografica finalizzata alla localizzazione della provenienza della roccia. La data di presa in consegna del reperto dovrà essere prontamente notificata a questo Ufficio e la riconsegna dovrà avvenire **entro due settimane da tale data**.

3. L'eventuale realizzazione e utilizzo di immagini (fotografie, scansioni 3D, radiografie, immagini TAC e ogni altra forma assimilabile) per fini di studio e di pubblicazione scientifica. Copia delle immagini realizzate durante lo studio dovrà essere consegnata alla Soprintendenza. Si ricorda inoltre che, ai sensi dell'art. 108 commi 3 e 3bis del D. Lgs 22.01.2004 n° 42 e ss.mm.ii., *Codice dei Beni Culturali e del Paesaggio*, nessun canone è dovuto per la riproduzione delle immagini per finalità di studio e ricerca. Si rimanda anche al Decreto del Soprintendente n.66/2018 contenente il regolamento per la fornitura, l'esecuzione e la riproduzione di immagini dei beni culturali in consegna alla Soprintendenza, consultabile sul sito web istituzionale.

Resta inteso che le indagini proposte avverranno senza oneri per questa Soprintendenza.

Si richiede, a studio concluso, una relazione scientifica e copia di ogni eventuale pubblicazione dei risultati ottenuti.

IL SOPRINTENDENTE
Vincenzo Tiné

Il Responsabile del Procedimento
Funzionario Antropologo Dott. Nico Radi
FORMAZIONE – Ufficio Formazione e Rapporti con l'Università
Telefono: 0102718221
E-mail: nico.radi@beniculturali.it

Il Funzionario Archeologo
Dott.ssa Marta Conventi
UTI 03.Area Savona Ovest
Telefono: 010 2718225
E-mail: marta.conventi@beniculturali.it
