# Supplementary material for: Neandertals on the beach: Use of marine resources at Grotta dei Moscerini (Latium, Italy)
Source: PLoS One. 2020 Jan 15;15(1):e0226690. doi: 10.1371/journal.pone.0226690 (PMC6961883; doi:10.1371/journal.pone.0226690)
Supplement: S4 File — (PDF) [file pone.0226690.s004.pdf]

Supplementary Information

Neandertals on the beach.

Use of marine resources at Grotta dei Moscerini (Latium, Italy)

Paola Villa*, Sylvain Soriano, Luca Pollarolo, Carlo Smriglio, Mario Gaeta,

Massimo D'Orazio, Jacopo Conforti, Carlo Tozzi

*** To whom correspondence should be addressed. E-mail: villap@colorado.edu**

S4 File. Permission to publish two photos by Barbara Wilkens.

Re: Permission to publish two photos from "Archeolozoologia. Manuale per lo studio dei resti faunistici dell'area mediterranea".

Barbara Wilkens <wilkensarcheozoologia@yahoo.com>
Wed 11/20/2019 12:07 PM

Cara Paola Villa

I confirm permission for the open access journal PLOS ONE to publish two partial photos of the internal face of Callista chione and Glycymeris glycymeris from my Manual, under the Creative Common Attribution License CC BY 4.0 (http://creativecommon.org/license/by/4.0/). I am aware that this license allows unrestricted use and distribution, even commercially, by third parties. The caption should say: Courtesy of Barbara Wilkens."

With all my thanks

Barbara Wilkens
