# Supplementary material for: Neandertals on the beach: Use of marine resources at Grotta dei Moscerini (Latium, Italy)
Source: PLoS One. 2020 Jan 15;15(1):e0226690. doi: 10.1371/journal.pone.0226690 (PMC6961883; doi:10.1371/journal.pone.0226690)
Supplement: S5 File — Exploratory experiment. (PDF) [file pone.0226690.s005.pdf]

Supplementary Information

Neandertals on the beach.
Use of marine resources at Grotta dei Moscerini (Latium, Italy)

**Paola Villa, Sylvain Soriano*, Luca Pollarolo, Carlo Smriglio, Mario Gaeta,
Massimo D'Orazio, Jacopo Conforti, Carlo Tozzi**

*** To whom correspondence should be addressed. E-mail:** sylvain.soriano@cnrs.fr

S5 File. Retouched shell tools. Exploratory experiment.

A small scale exploratory experiment was performed with the aim to evaluate the ease of shell retouching, the resharpening potential of shell tools and if the scalariform morphology of the retouch was a feature associated with resharpening or was due to the mineralogical structure of the shells. A large scale experimentation has already been performed by [1–3] but some of these questions were not examined or only in part. We hypothesize that the scalar morphology of retouch on *C. chione* shell tools is determined by the microstructure of the shell that control the development of fracture and not by the technique used to retouch the shells.

Shells of *C. chione* might have been preferred for their sturdiness, the morphology and size of the valves. Unlike some other species from the Veneridae family, *C. chione* is free from periostracal calcification (i.e. needles and pins) [4] so the outer surface of the shell is smooth. Thus the retouched valve will retain a regular edge. As demonstrated by studies relative to dredging damages, *C. chione* has a very strong and resistant shell compared to other species [5]. These discrepancies are explained by shell size, thickness, structure and microstructure. The interspecific variations of structure and microstructure of shells may conceivably be the cause for the response of shells to hammering to produce cutting tools by Neandertals.

The main component of bivalve shells consists of calcium carbonate and an organic matrix. The shell has a multi-layered structure with a periostracum (outermost layer) followed by outer, middle and inner layers. The microstructure of molluscan shells has been extensively described [6–11]. The microstructure of *C. chione* (name-bearing genus of Callistinae [12]) has not been fully described but a more general study of the Veneridae family is available [13]. In the Veneridae family, each layer (outer, middle and inner) is composed of minute aragonite crystals but the crystal forms and their arrangements are different in every species. For four species of the genus *Callista*, Shimamoto described the following combination: outer layer, crossed-lamellar structure; middle and inner layers, homogeneous structure. A homogeneous structure is an aggregation of granular crystals of various sizes and shapes. The change from a layer with a homogeneous structure and a layer with crossed-lamellar structure is normally gradual. The complex and multi-layered microstructure of bivalve shells is a means to stop cracks from forming or extending and is one of the three main components of the shell strength that allow resistance to breakage [14,15]. On radial sections of present-day of *C. chione* [16: fig. 2] one can observe that the thickness of middle/inner layers relative to the outer layer increases from the edge margin to the hinge. The decay of the organic matrix that initially protects the crystallites composing the mineral material of the shell induces a post-mortem reduction of the shell strength [15]. We do not know if a

difference in edge-wear resistance between fresh valves of *C. chione* and beached ones could have been noticed by Neandertals.

For this experiment, 65 valves of *C. chione* obtained from a retailer in Italy were cleaned by Carlo Smriglio. The valves were retouched by Sylvain Soriano on their ventral surface with the aim to obtain a sharp cutting edge similar to the archaeological shell tools. Different types of hammer, knapping motion and shell position were tested, including those described in Romagnoli et al. (2016), until a chaîne opératoire that induces the lowest fragmentation of the valves was found. This chaîne opératoire comprises two steps with a change of knapping technique between the first sharpening (Fig. 1) and the resharpening (Fig. 2). The initial edge of the valve is strengthened by its round cross-section. We have observed that this character increases the risk of breakage of the valve away from the margin when initially struck with a soft hammer (bone retoucher, soft-stone). Breakage was significantly reduced when a hard hammer was used for the initial sharpening. Resharpening, if any, was performed preferentially with a softer hammer, soft stone or bone retoucher that allows to obtain a more regularly retouched edge.

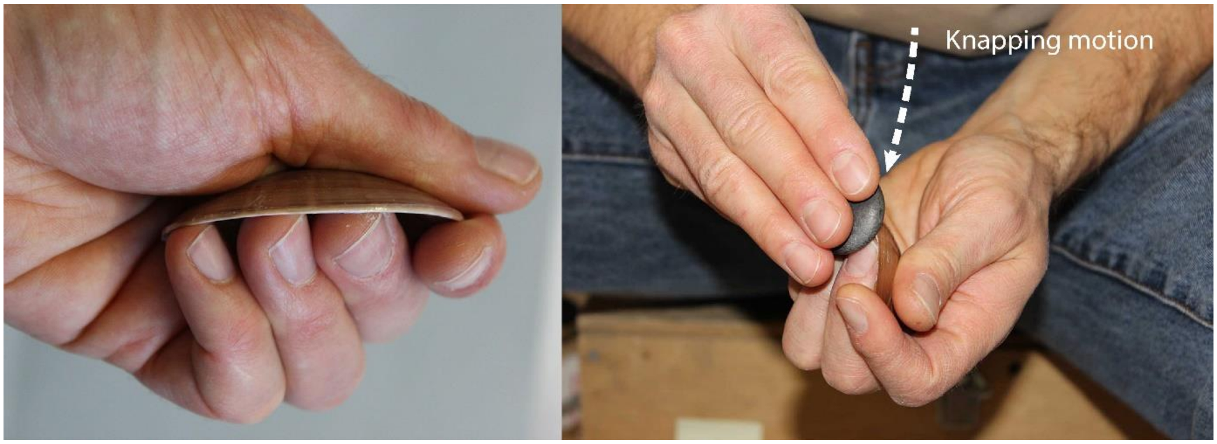

Figure 1. Holding the valve of *C. chione*, use of the hammer and knapping gesture for the initial sharpening (first row of retouch). Left: viewed from the eye of the knapper. Right: viewed from in front of the knapper. The valve is held vertically. The hammer (semi-hard stone) is a flat, elongated pebble of black shale (66 x 36 x 11 mm, weight 38 g). The knapping motion is almost vertical and is stopped immediately after the hit.

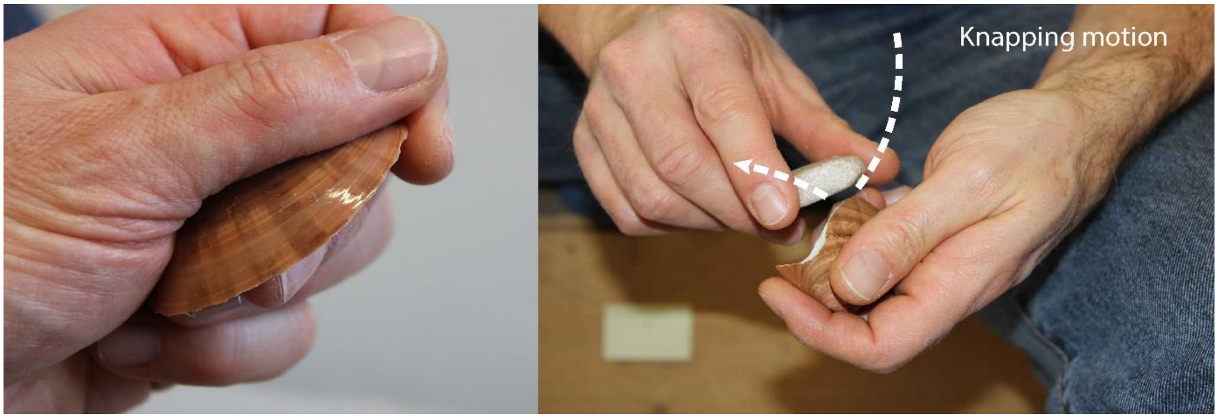

Figure 2. Holding the valve of *C. chione*, use of the hammer and knapping gesture for the resharpening phase (second row of retouch and following). Left: viewed from the eye of the knapper. Right: viewed from in front of the knapper. The valve is held obliquely, inclined toward the right of the knapper (if right-handed). The hammer (soft stone) is a flat quadrangular pebble of micritic limestone (38 x 35 x 14 mm, weight 35 g). A bone retoucher was also used with almost the same results. The knapping motion is curved and its direction changed after the hit (type "hit and back").

For each retouched portion of valve or fragment of valve the following criteria were recorded: type of knapping gesture, type of hammer, and morphology of the retouch. For this last feature we recorded the proximal morphology (morphology of the initiation point of the retouch in cross section) and the termination morphology. Depending on how the fracture is initiated in this mineral material (i.e. Hertzian or bending initiation [17,18]) one can observe a proximal cross section of the retouch on shells that is rectilinear (wedging-like), slightly concave (with Hertzian initiation) or slightly convex (with bending initiation). About the distal morphology of the retouch we distinguished those with an abrupt termination (stepped or hinged) from all other termination (feather, wavy…). Since important variations of retouch morphology along retouched edges were noticed, we distinguished and recorded for each retouched edge a dominant and a minor type, if any, for both initiation and termination morphology of retouch. The relationship of initiation and termination was examined (Table 1). Statistically, the null hypothesis is validated (Chi square=1.89, $p= 0.05$, df=2), the initiation type and termination types are unrelated variables. However, initiation type of retouch and hammer type are not independent variables (Table 2) (Exact Fisher test, p-value= 0.001122) as one can expect for knapped mineral materials. The more pronounced Hertzian initiation for the retouch was observed when the retouched edge was close to the umbo of the valve where inner/middle layers of the shell are thickest relative to outer layer (Fig. 3). In fact these layers are characterized by a more homogenous structure where a Hertzian cone fracture can progress more easily.

Table 1. Proximal and distal morphology of retouch on the experimental retouched *C. chione* shells.

	Termination type		
**Initiation type**	Stepped or hinged	Feather, wavy, other…	Total
Bending	7	8	15
Hertzian	24	17	41
Wedging-like	12	5	17
Total	43	30	73

Table 2. Proximal morphology of retouch relative to hammer type on the experimentally retouched *C. chione* shells.

	Hammer type			
**Initiation type**	Semi-hard, black shale	Soft stone, micritic limestone	Bone retoucher	Total
Bending	5	8	1	14
Hertzian	29	6	1	36
Wedging-like	6	2	4	12
Total	40	16	6	62

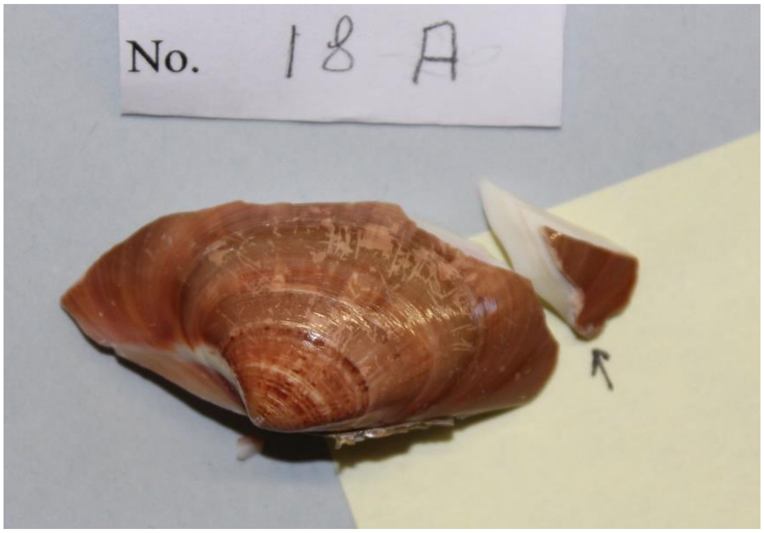

Figure 3. Very pronounced bulb (Hertzian initiation) indicated by the arrow on a retouch flake extracted from a retouched edge close to the umbo of the valve.

Two valves were each retouched three times (three sharpening) with the aim to examine the degree of overlap of the retouching rows. The extension of each row of retouch was marked by laying nail polish of different color before the next resharpening and recorded through photos. After the third sharpening phase, the edge is at most 2 mm away from the initial edge of the valve (Fig. 4: A). Due to roundness of the initial edge of the valve that forms a sort of striking platform, the edge retreat is almost null after the first sharpening. It is also noticeable that the range of the retouch from the third sharpening phase almost totally overlap retouch from the two previous ones (Fig. 4: B).

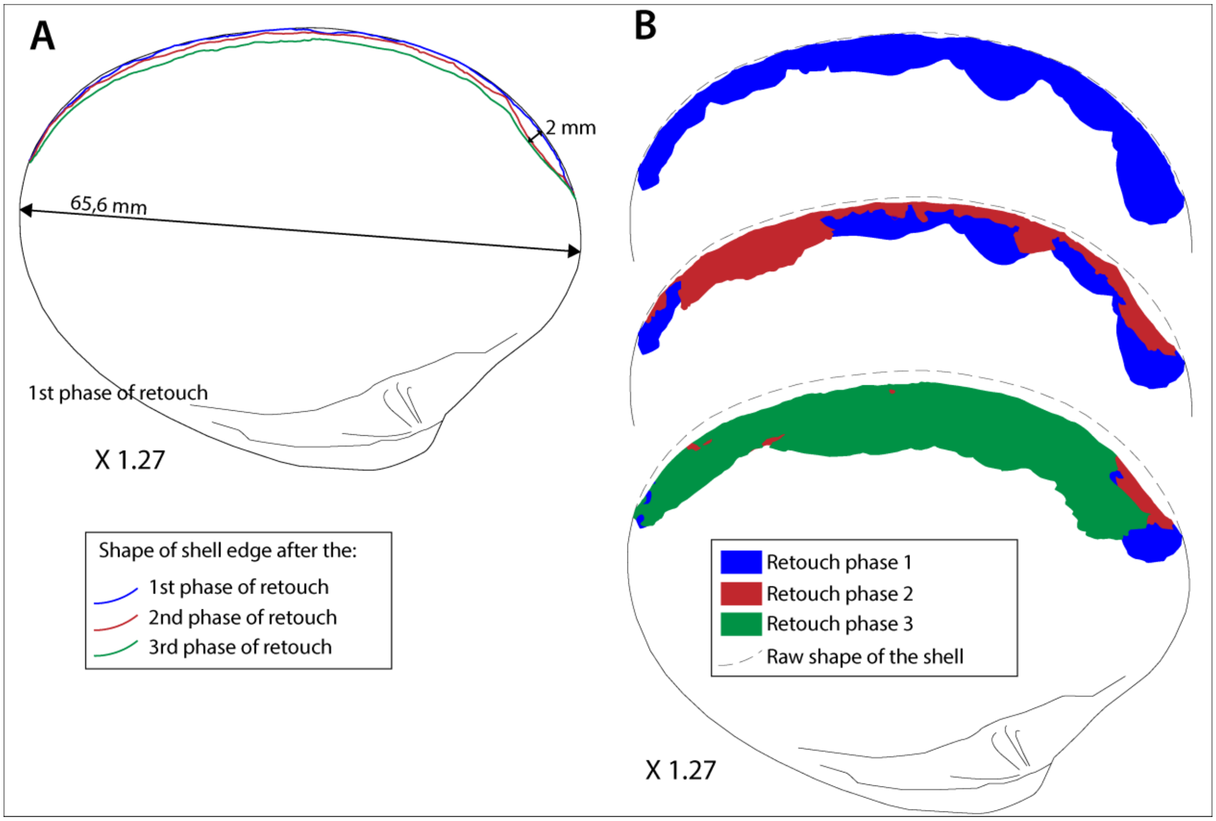

Figure 4. Record of the extension of retouch rows and edge shape of a *Callista chione* valve experimentally retouched. A: Color lines indicate the modification of edge shape after the first, the second and the third sharpening. B: Coloured areas represent the extension of the retouch from each sharpening. Same colour code as in A. The valve was 65.6 mm length and 56.6 mm width. Raw = natural.

Observations reported here need to be duplicated within the framework of a wider experiment but preliminary conclusions can be drawn.

C. chione shell tools can be resharpened many times before exhaustion of the shell. The very low thickness increase of the shell from the margin to the umbo ensures that the cutting wedge angle is maintained close to the initial one. In fact, both [19] and[2] observed a mean cutting wedge angle between 40 and 45° that is quite sharp (see [20] for a record of this feature on Acheulean flint flake tools). This is a major difference with flint tools on which it is almost impossible to maintain the same

cutting wedge angle through successive resharpening as the thickness of the blank usually rises from the initial edge toward the middle of the blank. From an economical point of view, shell tools might have been involved in activities requesting acute cutting edges and replace heavily retouched stone tools which can no more provide such cutting edges.

The scalariform (scaled) aspect of retouch on archaeological *C. chione* shell tools might be due to the mineralogical structure of the shell itself rather than to the superposition of several rows of retouch. The presence of retouch with stepped or hinged termination was noticed on 60% of experimentally retouched edges at the initial sharpening. Thus at this early stage of retouch the scalariform aspect cannot be explained by the succession of resharpening as described on Quina type scrapers on flint. Our observations also demonstrate that a resharpening can remove totally the negatives of previous rows of retouch. According to us, it is the complex microstructure of the shells, notably the change in orientation of crystal microstructures between outer and middle layer of the shell, that is responsible for the development of the scalariform aspect of retouch and that this aspect was not aimed at by Neandertal knappers.

References

1. Romagnoli F, Martini F, Sarti L. Neanderthal Use of Callista chione Shells as Raw Material for Retouched Tools in South-east Italy: Analysis of Grotta del Cavallo Layer L Assemblage with a New Methodology. J Archaeol Method Theory. 2015;22: 1007–1037. doi:10.1007/s10816-014-9215-x
2. Romagnoli F, Baena J, Sarti L. Neanderthal retouched shell tools and Quina economic and technical strategies: An integrated behaviour. Quaternary International. 2016;407: 29–44. doi:10.1016/j.quaint.2015.07.034
3. Romagnoli F, Baena J, Naranjo AIP, Sarti L. Evaluating the performance of the cutting edge of Neanderthal shell tools: A new experimental approach. Use, mode of operation, and strength of Callista chione from a behavioural, Quina perspective. Quaternary International. 2017;427: 216–228.
4. Glover EA, Taylor JD. Needles and pins: acicular crystalline periostracal calcification in venerid bivalves (Bivalvia: Veneridae). J Molluscan Stud. 2010;76: 157–179. doi:10.1093/mollus/eyp054
5. Vasconcelos P, Morgado-André A, Morgado-André C, Gaspar MB. Shell strength and fishing damage to the smooth clam (Callista chione): simulating impacts caused by bivalve dredging. ICES J Mar Sci. 2010; fsq149. doi:10.1093/icesjms/fsq149
6. Carter JG, Clark GR. Classification and Phylogenetic Significance of Molluscan Shell Microstructure. Studies in Geology, Notes for a Short Course. 1985;13: 50–71. doi:10.1017/S0271164800001093
7. Carter JG, Rhoads D, Lutz R. Guide to bivalve shell microstructures. Skeletal growth of aquatic organisms: biological records of environmental change. New York: Plenum Publishing Corporation; 1980. pp. 645–673.
8. de Paula SM, Silveira M. Studies on molluscan shells: Contributions from microscopic and analytical methods. Micron. 2009;40: 669–690. doi:10.1016/j.micron.2009.05.006

9. Chateigner D, Hedegaard C, Wenk H-R. Mollusc shell microstructures and crystallographic textures. Journal of Structural Geology. 2000;22: 1723–1735. doi:10.1016/S0191-8141(00)00088-2

10. Popov SV. Composite prismatic structure in bivalve shell. Acta Palaeontologica Polonica. 1986;31.

11. Popov SV. Formation of bivalve shells and their microstructure. Paleontol J. 2015;48: 1519–1531. doi:10.1134/S003103011414010X

12. Mikkelsen PM, Bieler R, Kappner I, Rawlings TA. Phylogeny of Veneroidea (Mollusca: Bivalvia) based on morphology and molecules. Zool J Linn Soc. 2006;148: 439–521. doi:10.1111/j.1096-3642.2006.00262.x

13. Shimamoto M. Shell Microstructure of the Veneridae (Bivalvia) and its Phylogenetic Implications. Sci Rep Tohoku University Second series, Geology. 1986;56: 1–40.

14. Currey JD. 8 - Shell Form and Strength. In: Trueman ER, Clarke MR, editors. Form and Function. San Diego: Academic Press; 1988. pp. 183–210. doi:10.1016/B978-0-12-751411-6.50015-1

15. Zuschin M, Stachowitsch M, Stanton RJ. Patterns and processes of shell fragmentation in modern and ancient marine environments. Earth-Science Reviews. 2003;63: 33–82. doi:10.1016/S0012-8252(03)00014-X

16. Ezgeta-Balic´ D, Peharda M, Richardson CA, Kuzmanic´M, Vrgoc N, Isajlovi I. Age, growth, and population structure of the smooth clam *Callista chione* in the eastern Adriatic Sea. Helgoland Marine Research 2011; 65: 457–465. DOI 10.1007/s10152-010-0235-y

17. Cotterell B, Kamminga J. The Formation of Flakes. Am Antiq. 1987;52: 675–708. doi:10.2307/281378

18. Prost D-C. Nouveaux termes pour une description microscopique des retouches et autres enlèvements. Bulletin de la Société Préhistorique Française. 1993;90: 190–195.

19. Cristiani E, Lemorini C, Martini F, Sarti L. Scrapers of Callista chione from Grotta del Cavallo (Middle Palaeolithic cave in Apulia): evaluating use-wear potential. In: Luik H, Choyke AM, Batey CE, Lõugas L, editors. From Hooves to Horns, from Mollusc to Mammoth - Manufacture and Use of Bone Artefacts from Prehistoric Times to the Present. Tallinn: Tallinn Book Printers; 2005. pp. 319–324.

20. Keeley LH. The Utilization of Lithic Artifacts. In: Singer R, Gladfelter BG, Wymer JJ, editors. The Lower Paleolithic site at Hoxne, England. Chicago and London: The University of Chicago Press; 1993. pp. 129–149.
